# Lung cancer cells expressing a shortened *CDK16* 3′UTR escape senescence through impaired miR‐485‐5p targeting

**DOI:** 10.1002/1878-0261.13125

**Published:** 2021-11-09

**Authors:** Qi Jia, Baiyun Xie, Zhaozhao Zhao, Leihuan Huang, Gang Wei, Ting Ni

**Affiliations:** ^1^ State Key Laboratory of Genetic Engineering and MOE Key Laboratory of Contemporary Anthropology Collaborative Innovation Center of Genetics and Development Human Phenome Institute School of Life Sciences and Huashan Hospital Fudan University Shanghai China

**Keywords:** 3′UTR, APA, cancer cell senescence, *CDK16*, miR‐485‐5p

## Abstract

Inducing senescence in cancer cells is an emerging strategy for cancer therapy. The dysregulation and mutation of genes encoding cyclin‐dependent kinases (CDKs) have been implicated in various human cancers. However, whether CDK can induce cancer cell senescence remains poorly understood. We observed that *CDK16* expression was high in multiple cancer types, including lung cancer, whereas various replicative senescence models displayed low *CDK16* expression. *CDK16* knockdown caused senescence‐associated phenotypes in lung cancer cell lines. Interestingly, the *CDK16* 3′ UTR was shortened in cancer and lengthened in senescence models, which was regulated by alternative polyadenylation (APA). The longer 3′UTR [using the distal polyA (pA) site] generated less protein than the shorter one (using the proximal pA site). Since microRNAs (miRNAs) usually bind to the 3′UTR of target genes to suppress their expression, we investigated whether miRNAs targeting the region between the shortened and longer 3′UTR are responsible for the reduced expression. We found that miR‐485‐5p targeted the 3′UTR between the distal and proximal pA site and caused senescence‐associated phenotypes by reducing protein production from the longer *CDK16* transcript. Of note, *CDK16* knockdown led to a reduced expression of MYC proto‐oncogene, bHLH transcription factor (*MYC*) and CD274 molecule (*PD‐L1*), which in turn enhanced the tumor‐suppressive effects of senescent cancer cells. The present study discovered that *CDK16*, whose expression is under the regulation of APA and miR‐485‐5p, is a potential target for prosenescence therapy for lung cancer.

## Graphical abstract

The present study discovers that non‐small cell lung cancer (NSCLC) and senescent cells have opposite trends in *CDK16* expression and alternative polyadenylation (APA) usage. APA‐mediated 3′ UTR shortening of *CDK16* in NSCLC cells evades miR‐485‐5p binding, resulting in increased *CDK16* expression. Moreover, both *CDK16* inhibition and miR‐485‐5p overexpression can induce senescence of lung cancer cells and thus promote tumor‐suppressive effects.

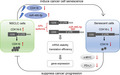

**Abbreviations**
APA: alternative polyadenylation
BLCA: bladder urothelial carcinoma
CCK8: cell counting kit‐8
CDK: cyclin‐dependent kinase
DEGs: differentially expressed genes
FC: fold change
GSEA: gene set enrichment analysis
HEK293T: human embryonic kidney 293T
HUVEC: human umbilical vein endothelial cells
KD: knockdown
LUAD: lung adenocarcinoma
LUSC: lung squamous cell carcinoma
miRNA: microRNA
NC: negative control
NSCLC: non‐small cell lung cancer
nt: nucleotide
OE: overexpress
pA: polyadenylation
PD‐L1: programmed cell death‐ligand 1
PDUI: Percentage of Distal poly(A) site Usage Index
PI: propidium iodide
qRT‐PCR: quantitative real‐time PCR
RBP: RNA binding protein
RNA‐seq: RNA sequencing
RPKM: Reads Per Kilobase per Million mapped reads
SA‐β‐Gal: senescence‐associated β‐galactosidase
shRNA: short hairpin RNA
TCGA: The Cancer Genome Atlas
UCEC: uterine corpus endometrial carcinoma

## 1 Introduction

Lung cancer is the most common cause of cancer death worldwide [1]. The majority of lung cancer patients are diagnosed with non‐small cell lung cancer (NSCLC), of which lung adenocarcinoma (LUAD) and lung squamous cell carcinoma (LUSC) are the most prevalent subtypes [2]. Therefore, it is of great clinical significance to explore the anticancer therapy for NSCLC. Cell cycle disorders, manifested as uncontrolled proliferation, are one of the common hallmarks in human cancers [3, 4]. Cell cycle progression is regulated by checkpoint controls and sequential activation of cyclin‐dependent kinases (CDKs), which contain 21 serine/threonine protein kinases in mammals [5, 6, 7]. Since CDK dysregulation and mutation have been implicated in human cancers, CDKs have been seen as potential therapeutic targets [3, 8, 9]. For example, pharmacologic inhibitors (such as palbociclib and abemaciclib) of CDKs 4 and 6 (CDK4/6) have shown significant inhibitory activities against several solid tumors, inducing G1 cell cycle arrest, causing quiescence, apoptosis, or senescence in various cancer cells [10, 11, 12]. Given that CDKs are essential for cancer cell survival and growth, it is of clinical significance to explore whether other CDKs play a role in NSCLC progression and treatment [13].

CDK16 (also known as PCTAIRE1) is a less studied member of the CDK family, but it is widely expressed in mammalian tissues [14] and highly expressed in a variety of cancer types, including prostate, breast, cervical, and lung cancers [15, 16]. CDK16 can promote cancer cell growth by phosphorylating tumor‐suppressor genes p27 and p53 to post‐translationally reduce their expression via the ubiquitin‐proteasome degradation pathway [15, 16, 17]. Moreover, CDK16 can promote tumor progression by regulating the mammalian target of rapamycin (mTOR) pathway [18]. In addition to its oncogenic roles, CDK16 has been implicated in other cellular processes, such as spermatogenesis and skeletal myogenesis [19, 20, 21]. Although *CDK16* is a proto‐oncogene with multiple cellular functions, it remains an open and critical question to determine its exact role in the progress of lung cancer development.

Cellular senescence is a biological process that leads to permanent cell cycle arrest [22] and can serve as a barrier to tumorigenesis [23]. Recently, inducing cancer cell to senescence has become a new and effective therapeutic strategy for cancer treatment in addition to apoptosis [24, 25]. Considering the involvement of CDK family members in cell cycle regulation, they are expected to play a role in cancer cell senescence induction. Indeed, this is the main mechanism of small molecule inhibitors of CDK4/6 in cancer treatment [10, 26]. Although previous studies have reported that *CDK16* is highly expressed in tumors and promotes cancer cell growth, whether it has a function in inducing cancer cell senescence remains unknown.

The mechanism by which CDK family members are dysregulated during cancer development is still not fully understood. The study of CDK regulation at post‐transcriptional level is largely lagging behind that at transcriptional level in diverse cancers. Alternative polyadenylation (APA) is a previously underestimated post‐transcriptional gene expression regulation but has recently attracted much attention in the study of diverse biological processes including cancer [27]. The majority of human genes have multiple polyadenylation (pA) sites, which may give rise to RNA isoforms with different 3′ ends [28, 29]. APA contributes to eukaryotic transcriptome diversification by generating transcript isoforms that differ in either coding sequence or 3′UTR [30, 31]. Furthermore, APA‐mediated gene expression regulation is widespread in a variety of human diseases such as cancer and in multiple biological processes such as cellular senescence [32, 33, 34], suggesting that APA of specific genes may be involved in inducing cancer cell senescence.

The 3′UTR can regulate gene expression by interacting with various trans‐acting factors, mainly miRNAs and RNA‐binding proteins (RBPs) [29]. Therefore, APA‐derived mRNA isoforms with different 3′UTR lengths can have different effects on mRNA metabolism, including RNA stability [35], localization, translation efficiency, and even protein localization [30, 36, 37, 38]. As widespread post‐transcriptional regulatory elements, miRNAs are a class of small non‐coding RNAs that negatively regulate target gene expression [39, 40]. Most mammalian genes are under the regulation of various miRNAs [41], and miRNAs have been implicated in various cellular processes and diseases, such as cell proliferation and cancer [39, 42]. Recently, the role of miRNAs has been disclosed in the progression of NSCLC. miRNAs have shown their tumor‐suppressive, oncogenic, diagnostic, and prognostic roles in lung cancer, and they can also be involved in regulating cancer cell metabolism and resistance or sensitivity of cancer cell to chemotherapy and radiotherapy [43, 44]. Moreover, some miRNAs can even serve as biomarkers for NSCLC diagnosis [45], such as miR‐504 [46] and miR‐21 [47, 48, 49], indicating the important functions of miRNAs in the development and progression of NSCLC. However, whether miRNA coordinating with 3′UTR changes caused by APA of its target gene can play a role in cancer cell senescence remains unclear.

The present study found that *CDK16* is a CDK undergoing APA, with 3′UTR shortening in four cancer types and lengthening in four cellular senescence models. *CDK16* transcript with longer 3′UTR generated less protein than that with shorter one. *CDK16* downregulation induced cellular senescence in two NSCLC cell lines, A549 and H1299. In addition, miR‐485‐5p can specifically bind to the alternative 3′UTR sequence of *CDK16*'s long transcript, which can explain the differential protein production between the two APA isoforms. *CDK16* knockdown also leads to a reduced expression of *MYC* and membrane programmed cell death‐ligand 1 (PD‐L1), two possible factors contributing to cancer cell senescence and immunotherapy effects, respectively [50, 51, 52]. In summary, *CDK16*, whose expression is regulated by APA in both cancer and senescence, is a potential novel therapeutic target for senescence‐mediated tumor suppression.

## 2 Materials and Methods

### 2.1 Cell culture and RNA interference

Four cell lines [A549, H1299, human embryonic kidney 293T (HEK293T), and human umbilical vein endothelial cells (HUVEC)] were originally obtained from the Cell Bank of the Chinese Academy of Sciences and available in our laboratory. Cells were all cultured in Dulbecco's modified Eagle medium (DMEM; Gibco, Thermo Fisher Scientific, Grand Island, NY, USA) supplemented with 10% (v/v) FBS in a humidified incubator (5% CO_2_) at 37 °C. Construction of a *CDK16* stable knockdown (KD) cell line was achieved by transfecting these cells with lentiviral short hairpin RNA (shRNA) specifically targeting *CDK16*, as well as the empty control vector pLKO.1. The clone IDs of shRNA were obtained from Sigma‐Aldrich as follows, shCDK16_#1: TRCN0000010251; shCDK16_#2: TRCN0000197222. Lentiviral vectors were constructed according to the established protocol on the Broad Institute RNAi Consortium (https://portals.broadinstitute.org/gpp/public/).

### 2.2 RNA extraction and quantitative reverse transcription PCR

Total RNA was extracted by TRIzol Reagent (Invitrogen, Thermo Fisher Scientific, Shanghai, China). cDNA synthesis was then performed with 500 ng DNA‐free total RNA using random hexamers and a FastQuant RT kit (Tiangen, Shanghai, China). Triplicate samples were subjected to quantitative reverse transcription PCR (qRT‐PCR) analysis using SYBR Green (Vazyme, Nanjing, China) to detect the expression of *CDK16* and its two isoforms with different 3′UTR length (CDK16‐S, CDK16‐L). For miRNA expression quantification, the miRNA 1st Strand cDNA Synthesis Kit (by stem‐loop; Vazyme #MR101) was used for reverse transcription. Then, miRNA Universal SYBR® qPCR Master Mix (Vazyme #MQ101) was used to amplify the expression of miR‐485‐5p, miR‐331‐3p, and miR‐3064‐5p. The primer sequences used were listed in Table S1.

Relative mRNA expression was calculated using the 2^‐∆∆Ct^ method [53]. *GAPDH* and *U6* were employed as an endogenous control for *CDK16* and miRNAs, respectively.

### 2.3 Western blot analysis

Total protein isolation was conducted utilizing T‐PER™ Tissue Protein Extraction Reagent (Thermo Fisher Scientific, Shanghai, China, Cat #78510). After centrifugation, the supernatant cell lysate was collected, then mixed with 4× loading buffer, and boiled for 10 min. Proteins were resolved in an SDS/PAGE gel (10%) and then transferred to PVDF membranes. Membranes were subjected to blocking, washing, antibody incubation, and detection by enhanced chemiluminescence. The antibodies used include CDK16 Rabbit pAb (PCTAIRE1 Polyclonal Antibody; Proteintech, Wuhan, China, Cat #10102‐1‐AP, at 1 : 600 dilution), GAPDH Rabbit mAb (Cell Signaling Technology, Boston, MA, USA, Cat #2118, at 1 : 1000 dilution), and c‐Myc Rabbit mAb (Cell Signaling Technology, Cat #18583, at 1 : 1000 dilution).

### 2.4 miRNA target prediction, plasmid construction, and miRNA transfection

miRNA target was predicted with TargetScan (http://www.targetscan.org/) [54] and TarBase [55] Specifically, we searched for miRNAs targeting *CDK16* in TargetScan Human 7.2, only allowing to show the conserved sites for miRNA families conserved only among mammals, and then, three miRNAs (miR‐485‐5p, miR‐331‐3p, and miR‐3064‐5p) were predicted to have binding potential to the 3′UTR of *CDK16*. It was also predicted by TarBase that *CDK16* is the target gene of miR‐485‐5p. The CDK16‐L and CDK16‐M plasmids were constructed by inserting the wild‐type and mutant 3′UTR sequence into the psiCHECK2 vector. The mimics and inhibitors of miR‐485‐5p, miR‐331‐3p, and miR‐3064‐5p and negative control (NC) were designed and synthesized by GenePharma (Shanghai, China). For transfection, miRNA mimics or inhibitors were transfected at 100 nm alone or in combination with the constructed plasmid (CDK16‐L or CDK16‐M) using Lipofectamine 2000 Transfection Reagent (Invitrogen) according to the manufacturer's manual.

### 2.5 Dual‐luciferase assay

To test the expression efficiency of the two APA isoforms of *CDK16*, short and long 3′UTR sequence were amplified from human genomic DNA and cloned into psiCHECK2 luciferase reporter vector using XhoⅠ and PmeⅠ restriction enzyme sites located at the 3′ end of the *Renilla* gene. The primers used to amplify CDK16‐S and CDK16‐L were as follows UTR‐Forward, 5′‐ccgctcgaggccacagaccgaggcccca‐3′, UTR‐S‐Reverse, 5′‐agctttgtttaaaccaagtgaaggagtgatgagagc‐3′, UTR‐L‐Reverse, 5′‐ agctttgtttaaacacagcgattatggtgcattc‐3′. Then, A549, H1299, HEK293T, and HUVEC cells plated in 24‐well plate were transfected with CDK16‐S and CDK16‐L constructs in four replicates and harvested after 48‐h cultivation. After lysing the cells with 100 μL Passive Lysis Buffer (1×), the activities of Firefly luciferase and *Renilla* luciferase in those cells were then detected by a microplate reader (TECAN, Shanghai, China) according to the standard protocol of a Dual‐Luciferase Reporter Assay System (Promega Biotech Co., Ltd., Beijing, China).

### 2.6 RNA stability assay

A549, H1299, HEK293T, and HUVEC cells were treated with 5 ng·mL^−1^ of Actinomycin D (Act D; Sigma‐Aldrich, Inc., St. Louis, MO, USA, A4262) for successive 0, 2, 4, 6, 8 h and harvested at each time point, and then, RNA was extracted and reverse transcribed into cDNA. The expression levels of transcripts with short and long 3′UTRs (CDK16‐S and CDK16‐L) were measured using qRT‐PCR with sequence‐specific primers at each time point.

### 2.7 Cell counting kit‐8 assay

A total of 100 μL cells were seeded in a 96‐well plate with at least 2000 cells per well. Then, 10 μL cell counting kit‐8 (CCK‐8) solution was added to each well, incubated for 2 h, and the absorbance at 450 nm was measured with a microplate reader (TECAN) every 24 h. The cell growth curve was drawn based on the absorbance value detected at each time point.

### 2.8 SA‐β‐Gal staining

Cells were seeded in 24‐well plate to grow to about 60% cell confluence 1 day in advance. Then, the standard procedure of senescence‐associated β‐galactosidase (SA‐β‐Gal) staining kit (Sigma‐Aldrich, Inc., Cat#: CS0030) was performed. After removing the culture medium, cells were washed twice with PBS (1×), then fixed for 7 min in fixation solution (1×), followed by three‐time washes with PBS, and then incubated overnight at 37 °C in fresh‐prepared staining buffer. The images were captured under a microscope (Leica, Wetzlar, Germany).

### 2.9 Flow cytometric analysis of cell cycle and apoptosis

For cell cycle assay, cells (A549, H1299, HEK293T, HUVEC) treated with shRNAs (shCDK16_#1, shCDK16_#2) and miR‐485‐5p mimic were collected separately, washed and resuspended in PBS (1×) containing 0.03% Triton X‐100 and 50 μg·mL^−1^ propidium iodide (PI). After staining in the dark for 10 min, cell cycle assay was performed using a BD Flow Cytometer. Cells transfected with 100 nm miR‐485‐5p mimic or NC were synchronized by 2.5 μm colchicine (MCE, Monmouth Junction, NJ, USA, Cat. No. HY‐16569) before cell cycle measurement. Cell apoptosis was assessed by a FITC‐Annexin V Apoptosis Detection Kit (BD BioSciences, San Jose, CA, USA, cat no.556547). Briefly, cells were collected, washed, and stained with FITC‐conjugated Annexin V and PI in the dark for 10 min, and then assayed by flow cytometry (FACS Calibur, BD BioSciences). The results of cell cycle and apoptosis assays were analyzed using modfit Lt 5.0 (Verify Software House, Topsham, ME, USA) and cellquest pro software (BD BioSciences), respectively. Each sample was tested for three times.

### 2.10 Membranous PD‐L1 detection

The protein expression of PD‐L1 in lung cancer cells (A549 and H1299) was determined by flow cytometry. *CDK16*‐KD cells were harvested, washed, resuspended in FACS Buffer (1% BSA in PBS), and then incubated in PBS containing 10% normal goat serum to block non‐specific protein–protein interactions. Cells were stained with 5 μg·mL^−1^ PE‐labeled anti‐PD‐L1 antibody (Abcam, Cambridge, UK, Cat # ab209962, at 1 : 100 dilution) for 30 min in the dark on ice. Then, the expression of PD‐L1 on cell surface was detected by flow cytometry and analyzed by cellquest pro software.

### 2.11 RNA sequencing library construction

Total RNA was extracted from *CDK16*‐KD A549 and H1299 cells. After capturing poly(A) mRNA from 1 μg purified total RNA, mRNA sequencing (or RNA‐Seq) libraries were constructed according to standard protocol of the KAPA Stranded mRNA‐Seq Kit and sequenced using Illumina HiSeq platform (Illumina, San Diego, CA, USA).

### 2.12 RNA‐seq data analysis

The raw paired‐end reads obtained from RNA sequencing (RNA‐seq) experiments were filtered to remove low‐quality reads using Trim Galore and then aligned to human reference genome sequence (UCSC hg19 assembly) using STAR with default settings [56]. To select genes with accurate expression value, we chose genes whose FPKM > 1 in at least one sample for subsequent analysis. Differential gene expression analysis was performed using EdgeR, and a statistical cutoff of FDR < 0.05 and fold change > 2 was applied to define differentially expressed genes (DEGs) [57]. Gene set enrichment analysis (GSEA) was performed by clusterProfiler [58], and hallmark gene sets (H collection) in the Molecular Signatures Database (MSigDB) were used for the GSEA [59].

### 2.13 Gene expression, APA, and survival analysis

The mRNA expression of *CDK16* in multiple cancer types was analyzed using the The Cancer Genome Atlas (TCGA) datasets from Gene Expression Profiling Interactive Analysis (GEPIA) website and quantified as log_2_(TPM + 1) [60], and the *CDK16* expression based on public RNA‐seq datasets of human senescent cells and matched young cells, including BJ, WI‐38, human foreskin fibroblasts (HFF), and MRC_5 [61], was indicated by Reads Per Kilobase per Million mapped reads (RPKM). For APA analysis, Dynamic analysis of APA from RNA‐seq (DaPars) [62] was used to identify the significantly changed APA events between two conditions (Tumor vs Normal; Senescence vs Young), and the resulted Percentage of Distal poly(A) site Usage Index (PDUI) value was used to indicate the percentage of transcripts using the distal poly(A) site. The APA usage change for a give gene between two conditions was quantified as a change in PDUI (ΔPDUI), which reflects the relative lengthening (positive index) or shortening (negative index) of 3′UTRs. We also used another algorithm to analyze the APA events of *CDK16*, that is, the RUD method. Specifically, two pA sites with the highest PSE (percentage of samples with expression) in the PolyA_DB database (version 3.2) [63] were extracted as the proximal and distal pA sites of *CDK16* and used for RUD calculation, during which process, the constitutive 3′UTR (cUTR) was defined as the region between stop codon to proximal pA site, and the alternative 3′UTR (aUTR) was defined as the region from proximal pA site to distal PA site. For survival analysis, we conducted the overall survival analysis based on *CDK16* expression on GEPIA platform. We used the Quartile group method and define patients with the top 25% *CDK16* expression level as high‐expression group and the lowest 25% as the low‐expression group. Then, Kaplan–Meier survival plot stratified by *CDK16* expression was plotted, with difference significance (*P*‐value) calculated using the log‐rank test.

### 2.14 Statistical analysis

All results were represented as the mean ± SEM (standard error of mean) of at least three independent experiments. All figures and statistics were generated by graphpad prism (GraphPad Software, Inc., San Diego, CA, USA). Unpaired *t*‐test was used for comparison between groups. *P* value < 0.05 was considered to be statistically significant. *, **, and *** represent *P* < 0.05, represents *P* < 0.01 and *P* < 0.001, respectively.

## 3 Results

### 3.1 *CDK16* has opposite trend in expression and 3′UTR length changes in cancer and aging processes

**Fig. 1:**
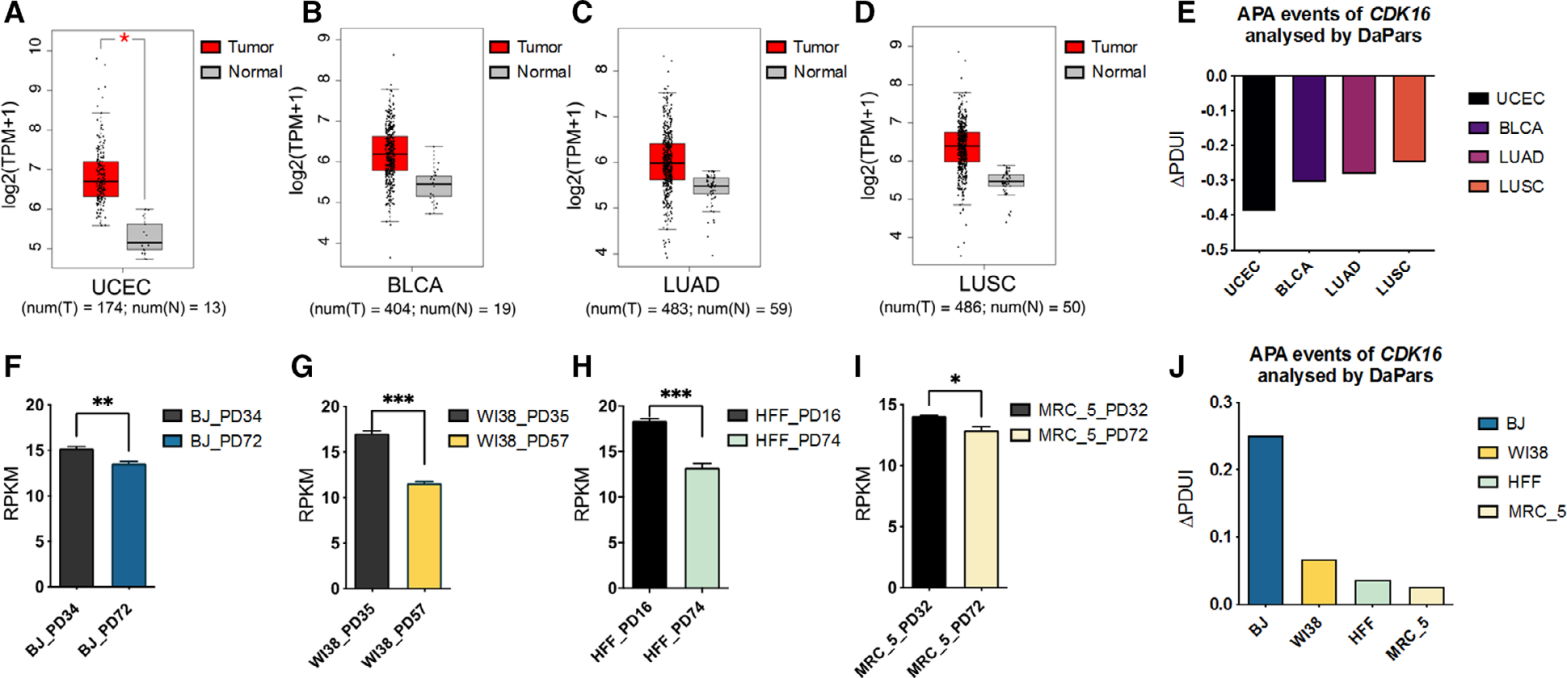
The expression pattern and 3′UTR length changes of *CDK16* in cancer and senescence. (A) Higher *CDK16* expression in UCEC tumors compared with matched normal tissues based on TCGA RNA‐seq datasets archived in GEPIA database [60]. The red and grey boxes represent the *CDK16* expression levels in TCGA tumors (T) and matched TCGA normal tissues (N), respectively. The expression level was displayed as log_2_(TPM + 1) denoted in Y‐axis. The number (num) of samples was marked below the graph, num(T) = 173, num(N) = 13. * represents *P* < 0.05 based on one‐way ANOVA. (B) Higher *CDK16* expression in BLCA tumors compared with matched normal tissues. num(T) = 404, num(N) = 19. (C) Higher *CDK16* expression in LUAD compared with matched normal tissues. num(T) = 483, num(N) = 59. (D) Higher *CDK16* expression in LUSC compared with matched normal tissues. num(T) = 486, num(N) = 50. The horizontal lines within each box represent the median values, the box spans the first quartile to the third quartile (interquartile range, IQR), and the whiskers represent 1.5 × IQR. (E) APA‐mediated 3′UTR shortening of *CDK16* in four cancer types compared to matched normal tissues based on DaPars analysis on public RNA‐seq data [62]. Y‐axis stands for the APA usage changes denoted by ΔPDUI. ΔPDUI value was calculated by subtracting the PDUI value in normal tissues from the PDUI value in each cancer type. (F) Reduced *CDK16* expression in senescent human fibroblasts BJ (Population Doubling 72, PD72) compared to corresponding young cells (PD34) based on public RNA‐seq datasets [61]. The expression level was indicated by RPKM. (G) Reduced *CDK16* expression in senescent (PD57) compared with young (PD35) human embryonic lung fibroblasts WI38. (H) Reduced expression of *CDK16* in HFF at PD74 than that at PD16. (I) Decreased *CDK16* expression in PD72 of human lung fibroblasts MRC_5 than in PD32. For panel F–I, unpaired *t*‐test was performed based on three biological replicates. *, **, *** represent *P* < 0.05, *P* < 0.01, *P* < 0.001, respectively. Error bars indicated mean ± SEM. (J) APA‐mediated 3′UTR lengthening of *CDK16* in the four human senescent cells compared to young cells based on public RNA‐seq data mentioned above. The positive ΔPDUI value quantified by DaPars represents 3′UTR lengthening in senescent cells compared to relatively young cells.

Since *CDK16* is the only CDK member with APA in the 3′UTR based on DaPars method [62], we are curious whether the poly(A) site usage changes in tumors comparing to normal tissues. The change in PDUI (percentage of distal polyA site usage index; ΔPDUI) calculated by DaPars [62] was used for evaluating pA site usage change in multiple cancer types, including uterine corpus endometrial carcinoma (UCEC), bladder urothelial carcinoma (BLCA), LUAD, and LUSC, along with corresponding matched normal tissues. Interestingly, *CDK16* was highly expressed (Fig.1A–D) and preferred proximal poly(A) site (indicated by negative ΔPDUI values; Fig. 1E) in these four cancer types, indicating the potential association between upregulated gene expression and shortened 3′UTR of *CDK16* in tumors, which is in line with previous observations of global 3′UTR shortening and related expression changes in multiple cancers [35]. Noteworthy, the same conclusion can also be drawn by using the RUD (relative usage of distal pA site) method [64] (Fig. S1A). Moreover, the preference of proximal *CDK16* pA site in lung cancer (LUAD and LUSC) was further verified using corresponding RNA‐seq tracks achieved in TCGA database (Fig. S2A). Considering the potential anti‐tumor effects of cancer cell senescence, we also surveyed the *CDK16* mRNA level in multiple senescence cells and found that *CDK16* showed decreased expression in four senescent cell types, including human primary fibroblasts (BJ), human embryonic lung fibroblasts (WI38), HFF, and human embryonic lung fibroblasts (MRC_5; Fig.1F–I), based on their public RNA‐sequencing (RNA‐seq) datasets [61]. We then analyzed the APA usage changes in these senescence models using ΔPDUI by DaPars and found that the distal poly(A) site was preferred (indicated by positive ΔPDUI values) in all senescence cells (Fig. 1J), suggesting that *CDK16* has a longer 3′UTR in aging cells than in young cells. However, there was no significant difference between the RUD values of young and senescent cells (Fig. S1B). In summary, *CDK16* mRNA levels and APA‐caused 3′UTR length changes had opposite trends in cancer and cellular aging processes.

### 3.2 *CDK16* downregulation induces senescence in two lung cancer cell lines

**Fig. 2:**
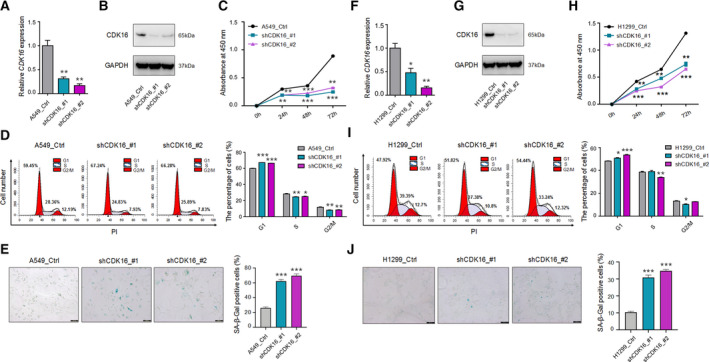
*CDK16* downregulation induces senescence in NSCLC cell lines A549 and H1299. (A, B) The validation of *CDK16* KD in A549 with two shRNAs (shCDK16_#1, shCDK16_#2) assayed by qRT‐PCR (A) and western blot (B). *GAPDH* serves as the internal control. (C) Cell proliferation rate of *CDK16*‐KD A549 cells evaluated by CCK‐8 assay. (D) Cell cycle analysis on *CDK16*‐KD A549 cells, cell cycle arrested at G1 phase in *CDK16*‐KD cells was measured by flow cytometry, shown as cell proportion at each phase (Left) and the statistical results (Right). (E) SA‐β‐Gal staining of *CDK16*‐KD and control A549 cells (Left) and the statistical results of the staining positive cells (Right). Scale bars = 200 μm. *** represents *P* < 0.001 based on *t*‐test with three independent countings. (F, G) Validation of *CDK16* KD in H1299 cells by qRT‐PCR (F) and western blot (G). *GAPDH* serves as the internal control. (H) Cell proliferation rate evaluated by CCK‐8 assay in *CDK16*‐KD H1299 cells. (I) Cell cycle analysis on *CDK16*‐KD H1299 cells detected by flow cytometry. The proportion of cells in each phase was shown by the distribution (Left) and the quantitative statistics (Right). (J) SA‐β‐Gal staining in *CDK16*‐KD and control H1299 cells (Left) and the quantitative statistics of staining positive cells (Right). Scale bars = 200 μm. For each panel, *, ** and *** stand for *P* < 0.05, *P* < 0.01, and *P* < 0.001, respectively, based on *t*‐test with three biological replicates. Error bars indicated mean ± SEM.

As demonstrated above, *CDK16* is highly expressed in lung cancer (LUAD and LUSC) and lowly expressed in senescent human embryonic lung cells (WI38 and MRC_5). Intriguingly, LUAD patients with high *CDK16* expression have a lower survival rate compared with those with low *CDK16* expression (Fig. S2B), which suggests that *CDK16* probably plays a role in lung cancer progress. To examine the possible roles of *CDK16* in lung cancer cells, we KD *CDK16* in two LUAD ‐related cell lines, A549 and H1299. Efficient *CDK16* KD in A549 cells using two shRNA (shCDK16_#1, shCDK16_#2) was confirmed (Fig. 2A,B). *CDK16*‐KD cells showed decreased cell proliferation rate compared with control (A549_Ctrl) cells, as detected by CCK‐8 assay (Fig. 2C). In addition, the percentage of G1‐phase cells were significantly increased in *CDK16*‐KD A549 cells compared with control cells (Fig. 2D). Importantly, *CDK16*‐KD A549 cells also showed a higher occurrence percentage of positive SA‐β‐gal staining, which has been regarded as a classical senescence marker [65] (Fig. 2E). These results indicated that *CDK16* inhibition gave rise to a series of senescence‐associated phenotypes in A549 cells.

Of note, A549 cells have wild‐type p53, while H1299 cells are p53‐deficient. The present study therefore explored whether *CDK16*‐KD‐induced cancer cell senescence is p53‐dependent or not. Consistent with the results in A549 cells, *CDK16‐*KD H1299 cells showed a series of senescence‐associated phenotypes, including decreased cell proliferation rate, cell cycle arrest in G1 phase, and more SA‐β‐gal‐positive staining cells compared with control cells (H1299_Ctrl; Fig. 2F–J). These above results indicated that senescence‐associated phenotypes in *CDK16*‐KD lung cancer cells were independent of p53, at least in H1299 cells. In addition, *CDK16‐*KD using two shRNAs was also performed in other two cell types (HEK293T and HUVEC). Similarly, *CDK16*‐KD resulted in senescence‐associated phenotypes (Fig. S3A–F,I–N) as observed in lung cancer cells. Moreover, *CDK16*‐KD also caused apoptotic phenotypes in normal HEK293T and HUVEC (Fig. S3G,H,O,P). Therefore, *CDK16* inhibition can induce senescence in both cancer and normal cells, suggesting a universal role of *CDK16* in cellular senescence.

### 3.3 *CDK16*‐L transcript has lower protein production than *CDK16*‐S transcript

**Fig. 3:**
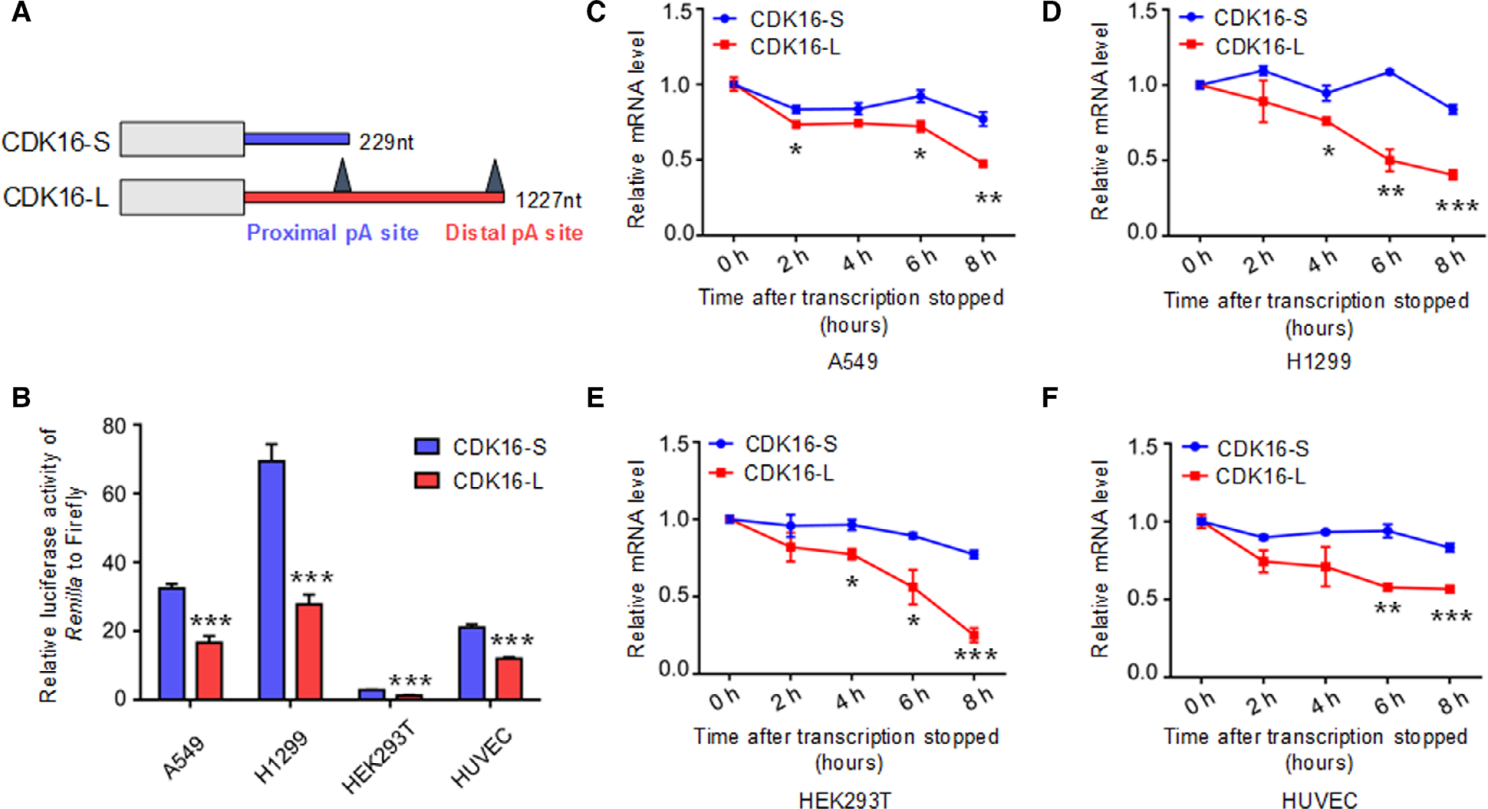
CDK16‐L has an inhibitory effect on gene expression compared with CDK16‐S. (A) Schematic diagram of mRNA isoforms with different 3′UTR length. CDK16‐S and CDK16‐L represent transcript of *CDK16* with short (226 nt) and long 3′UTR (1227 nt), respectively. The pA (polyA) sites were also shown in the 3′UTR. (B) Dual‐luciferase reporter assay to test the influence of 3′UTRs (CDK16‐S and CDK16‐L) on the luciferase activity in A549, H1299, HEK293T, and HUVEC. Relative luminescence of *Renilla* luciferase was normalized using the reference Firefly luciferase activity. *** represents *P* < 0.001 in *t*‐test with four biological replicates. Error bars indicated mean ± SEM. (C–F) The degradation rate of transcripts with different 3′UTR lengths measured by qRT‐PCR after blocking transcription by Actinomycin D. Relative mRNA level of *CDK16‐S* and *CDK16‐L* at each different time point was compared to 0 h in A549 (C), H1299 (D), HEK293T (E), and HUVEC (F). For panel C–F, *, **, and *** stand for *P* < 0.05, *P* < 0.01, and *P* < 0.001, respectively, based on *t*‐test with three biological replicates. Error bars indicated mean ± SEM.

Since the longer 3′UTR is associated with low *CDK16* expression and *CDK16* downregulation can induce senescence‐associated phenotypes, it was thus hypothesized that APA‐mediated 3′UTR lengthening in *CDK16* contributes to its decreased expression. According to the polyA database in the UCSC Genome Browser [66], *CDK16* has two pA sites in the 3′UTR, resulting in two transcripts with different 3′UTR lengths (short: CDK16‐S, long: CDK16‐L; Fig. S4A,B; Fig. 3A). To test the effects of these APA isoforms on gene expression, these two 3′UTRs of different lengths were respectively cloned into a dual‐luciferase reporter vector, wherein, the *Renilla* luciferase fluorescence intensity was normalized to that of Firefly luciferase. The results showed that the reporter gene with CDK16‐L produced less luciferase activity than that with CDK16‐S in both lung cancer cell lines (A549 and H1299) and normal cells (HEK293T and HUVEC; Fig. 3B). And mRNA stability testing of these two APA isoforms indicated that CDK16‐L is more susceptible to degradation than CDK16‐S in all of the tested cells (Fig. 3C–F), consistent with the argument that different 3′UTR lengths can affect mRNA degradation in various ways [67, 68]. To further validate the effects of different 3′UTR lengths on the protein production, we constructed two plasmids, containing the long and short 3′UTR fused with GFP tag, respectively (Fig. S5A). The results of Western blot showed that GFP protein expression in fusion with CDK16‐L was significantly decreased compared to that with CDK16‐S in both lung cancer cell lines (A549 and H1299) and normal cells (HEK293T and HUVEC; Fig. S5B–E). Therefore, APA‐mediated 3′UTR length changes explain, at least in part, the opposite gene expression of *CDK16* in cancer and senescence models.

### 3.4 miR‐485‐5p is responsible for inhibitory effect in alternative *CDK16* 3′UTR

To determine which regulatory elements in the alternative 3′UTR caused the expression differences between these two *CDK16* isoforms, four truncated reporter plasmids containing different alternative 3′UTR length [CDK16‐T1, CDK16‐T2, CDK16‐T3, and CDK16‐T4, in a 200 nucleotide (nt) length gradient] were constructed (Fig. S6A). By comparing relative luciferase activities of these constructs to CDK16‐S, CDK16‐T1 showed the most dramatic signal drop in all four tested cell lines (A549, H1299, HEK293T, and HUVEC; Fig. S6B–E), indicating that the sequence between CDK16‐T1 and CDK16‐S likely harbors the main inhibitory elements.

Since miRNAs usually bind to the 3′UTR of target genes to suppress their expression [69, 70, 71], we wondered whether miRNAs targeting this region (between CDK16‐S and CDK16‐T1) are responsible for the reduced *CDK16* expression. Three miRNAs (miR‐3064‐5p, miR‐485‐5p, and miR‐331‐3p) were predicted to specifically target this region by TargetScan [54, 72] (Fig. S7A). We first examined whether these miRNAs expressed in the above four cell lines, the results showed that all of them had detectable expression in the tested cells (Fig. S7B). To validate which miRNA represses *CDK16* expression, Mimics of these three miRNAs and NC were separately cotransfected with luciferase reporter plasmid containing CDK16‐L into HEK293T cells. The result showed that overexpression of miR‐3064‐5p and miR‐485‐5p reduced the relative luciferase activities of CDK16‐L construct (Fig. S7C). To further confirm the inhibitory effect of these two miRNAs, corresponding anti‐miRNA oligonucleotides were cotransfected with CDK16‐L, and the result showed that the relative luciferase activity was increased only when miR‐485‐5p was inhibited (Fig. S7D,E). These above results suggest that miR‐485‐5p is the main contributor to decreased expression of CDK16‐L comparing to CDK16‐S.

**Fig. 4:**
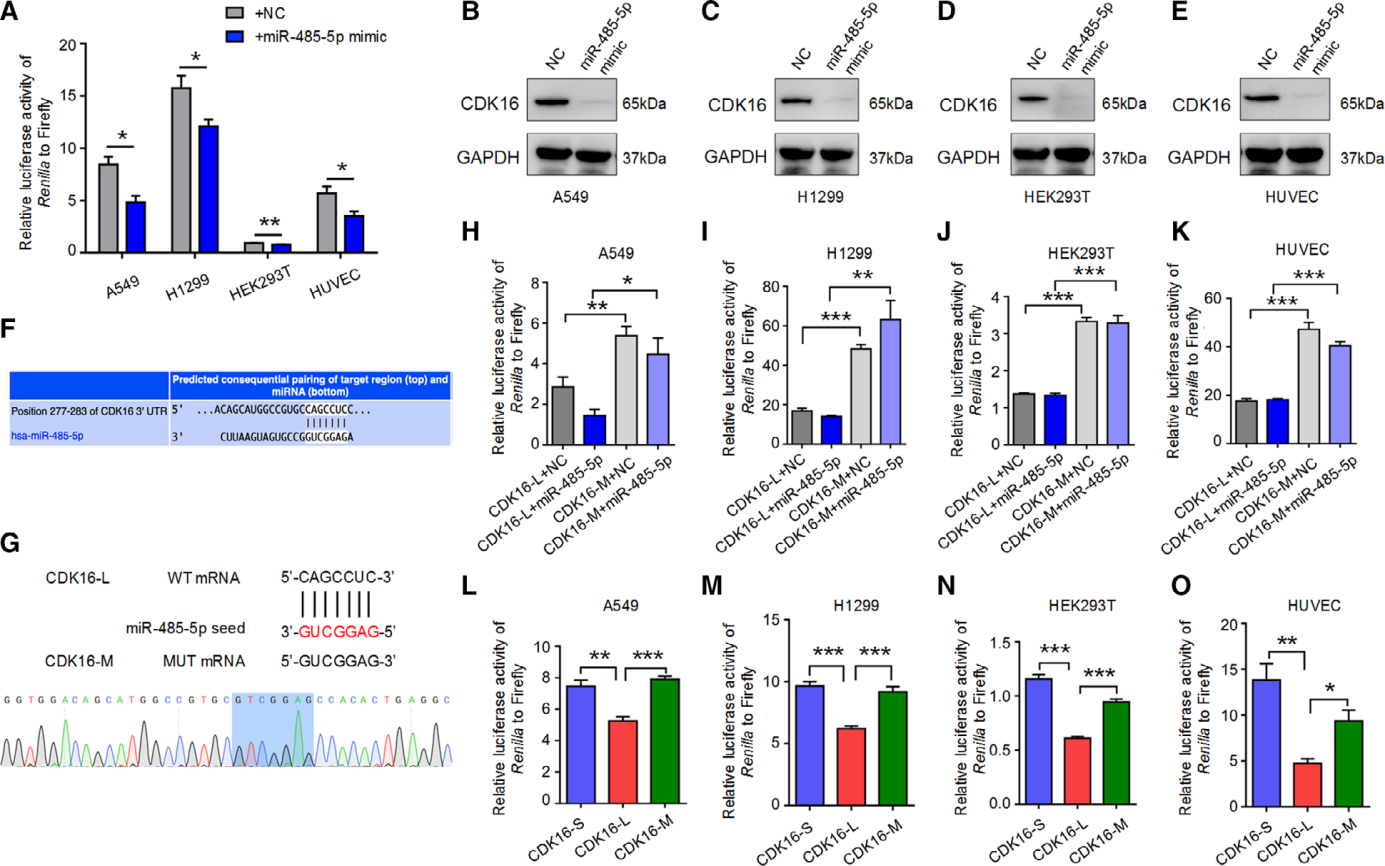
miR‐485‐5p is responsible for the reduced protein production of CDK16‐L. (A) Dual‐luciferase reporter assay to test the effect of miR‐485‐5p on the luciferase activity derived from CDK16‐L. After cotransfecting CDK16‐L with either miR‐485‐5p mimic or NC into A549, H1299, HEK293T, and HUVEC cells, *Renilla* luciferase activity relative to Firefly luciferase activity was measured. Error bars indicated mean ± SEM. *, ** represent *P* < 0.05 and *P* < 0.01, respectively, based on *t*‐test with four biological replicates. (B–E) Western blot assay to validate the decreased CDK16 protein expression in miR‐485‐5p‐overexpressing A549 (B), H1299 (C), HEK293T (D), and HUVEC cells (E). GAPDH serves as a loading control. (F) Prediction of *CDK16* as a target gene of miR‐485‐5p using TargetScan [54]. The binding site was located in the alternative 3′UTR of *CDK16*. (G) Mutation of miR‐485‐5p binding site. Based on the seed sequence of miR‐485‐5p, a fully noncomplementary mutant vector (CDK16‐M) was constructed and verified by Sanger sequencing. (H–K) Dual‐luciferase reporter assay to evaluate the relative luciferase activity of CDK16‐M. CDK16‐L or CDK16‐M was cotransfected with either miR‐485‐5p mimic or the NC into A549 (H), H1299 (I), HEK293T (J), and HUVEC cells (K), respectively. Error bars indicated mean ± SEM. *, **, *** represent *P* < 0.05, *P* < 0.01, and *P* < 0.001, respectively, based on *t*‐test with four biological replicates. (L–O) Dual‐luciferase reporter assay to evaluate the relative luciferase activity of CDK16‐S, CDK16‐L, and CDK16‐M in A549 (L), H1299 (M), HEK293T (N), and HUVEC (O). *, **, *** represent *P* < 0.05, *P* < 0.01, and *P* < 0.001, respectively, based on *t*‐test with four biological replicates. Error bars indicated mean ± SEM.

Of note, miR‐485‐5p binding to the 3′UTR of *CDK16* can also be predicted by Tarbase (Fig. S7F), which contains the experimentally supported miRNA‐mRNA interaction information [55], suggesting that *CDK16* is probably a target gene of miR‐485‐5p. To further verify the effect of miR‐485‐5p on the expression of *CDK16* longer isoform, the dual‐luciferase reporter assay was performed after cotransfecting CDK16‐L with either a miR‐485‐5p mimic or a NC into the above four cell lines (A549, H1299, HEK293T, and HUVEC). The results showed that miR‐485‐5p overexpression significantly reduced the relative luciferase activity of CDK16‐L isoform in both lung cancer and normal cells (Fig. 4A). Furthermore, miR‐485‐5p overexpression could reduce the CDK16 protein expression, as detected by Western blot (Fig. 4B–E). According to TargetScan, the 7 nt seed sequence of miR‐485‐5p is completely complementary to the target sequence in the alternative 3′UTR of *CDK16* (Fig. 4F). Therefore, it was hypothesized that these 7‐nt sequence is critical for the miRNA‐mediated decreased expression. To confirm this hypothesis, we mutated the potential miR‐485‐5p binding sequence on CDK16‐L (termed as CDK16‐M; Fig. 4G) to see if it affects miR‐485‐5p binding. By cotransfecting the miR‐485‐5p mimic or NC with CDK16‐M or CDK16‐L into the four cell lines used above (A549, H1299, HEK293T, and HUVEC), we found that CDK16‐M usually had a higher relative luciferase activity than CDK16‐L (Fig. 4H–K). Importantly, CDK16‐M was able to fully or partially rescue the reduced CDK16‐L luciferase activity in the above four cell types (Fig. 4L–O). These results above strongly support that miR‐485‐5p is a key regulator repressing the expression of longer transcript of *CDK16* using the distal pA site and can also explain the potential mechanism of APA regulating *CDK16* expression at the post‐transcriptional level.

### 3.5 miR‐485‐5p mimic promoted senescence‐associated phenotypes in two lung cancer cells

**Fig. 5:**
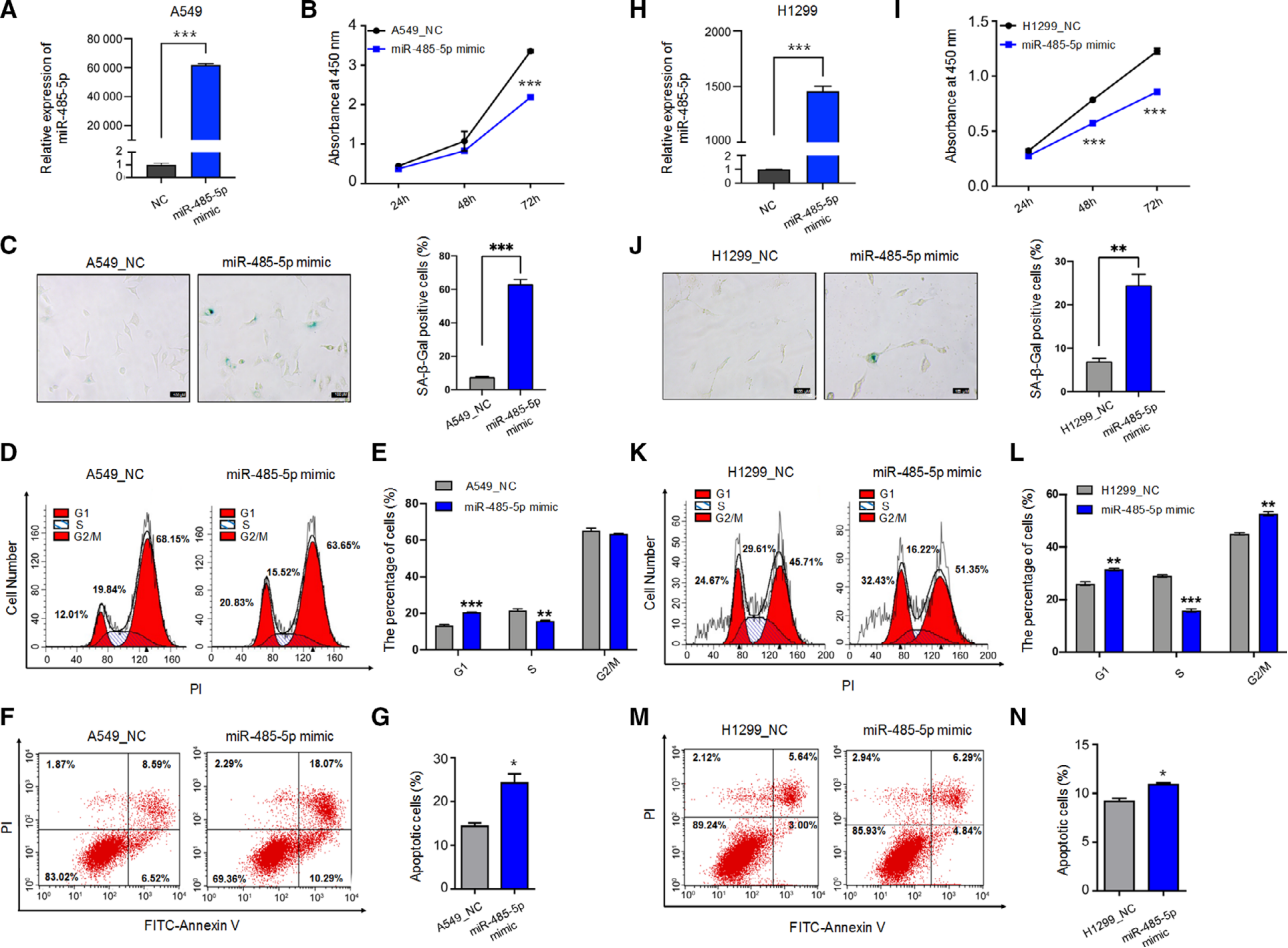
miR‐485‐5p has a positive effect on senescence and apoptosis in both A549 and H1299. (A) qRT‐PCR to test the expression of miR‐485‐5p in A549 cells transfected with miR‐485‐5p mimic and the NC. *U6* serves as the internal control. (B) CCK‐8 assay in miR‐485‐5p‐overexpressing (OE) A549 cells. (C) SA‐β‐Gal staining in miR‐485‐5p‐ OE A549 cells. Images were captured at 100× magnification under a microscope (Left) and the percentage of staining positive cells was quantified (Right). Scale bars = 100 μm. *** represents *P* < 0.001 based on *t*‐test with three independent countings. (D, E) Cell cycle analysis showing flow cytometric distribution (D) and quantitative statistics (E) for cells of each cell phase. Cells were synchronized by 2.5 μm colchicine before transfection and cell cycle measurement. (F, G) Apoptosis analysis with double‐staining Annexin V and PI in miR‐485‐5p‐OE A549 cells by flow cytometer (F). Annexin V‐positive cells were considered as apoptotic cells (G). (H) qRT‐PCR to validate the increased expression of miR‐485‐5p after transfecting its mimic into H1299 cells. *U6* serves as the internal control. (I) CCK‐8 assay in miR‐485‐5p‐OE H1299 cells. (J) SA‐β‐Gal staining in miR‐485‐5p‐OE H1299 cells. Images were captured at 100× magnification under a microscope (Left) and the percentage of positive staining cells was quantified (Right). Scale bars = 100 μm. ** represents *P* < 0.01 based on *t*‐test with three independent countings. (K, L) Cell cycle analysis shown by flow cytometric distribution (K) and quantitative statistics (L) for cells of each phase. Cells were synchronized by 2.5 μm colchicine before transfection and cell cycle measurement. (M, N) Apoptosis analysis by double‐staining Annexin V and PI in miR‐485‐5p‐OE H1299 cells by flow cytometer (M). The proportion of apoptotic cells is indicated by the percentage Annexin V‐positive cells (N). For each panel, *, **, *** represent *P* < 0.05, *P* < 0.01, and *P* < 0.001, respectively, based on *t*‐test with three biological replicates. Error bars indicated mean ± SEM.

Since decreased *CDK16* expression associated with its distal poly(A) site usage can lead to cellular senescence in lung cancer cells, and miR‐485‐5p can suppress the expression of longer transcript of *CDK16*, it was speculated that miR‐485‐5p has the potential to promote senescence in the case of the considerable distal pA site usage. To test this, the miR‐485‐5p mimic and NC were transfected into two lung cancer cell lines (A549 and H1299; Fig. 5A,H). miR‐485‐5p overexpression resulted in senescence‐associated phenotypes, including reduced cell proliferation rate (Fig. 5B,I), increased percentage of positive SA‐β‐gal‐staining cells (Fig. 5C,J), and cells arrested at G1 cell cycle phase (Fig. 5D,E,K,L) in both A549 and H1299 cells, similar to the phenomena observed in *CDK16*‐KD cells. These results above indicate that miR‐485‐5p can induce senescence‐associated phenotypes in lung cancer cells.

*CDK16* depletion in lung cancer has been reported to promote apoptosis, another tumor‐suppressive mechanism in addition to cellular senescence [16], so we wondered whether miR‐485‐5p can also induce apoptosis in lung cancer cells. Not surprisingly, more apoptotic cells were observed in A549 and H1299 cells transfected with miR‐485‐5p mimic (Fig. 5F,G,M,N), suggesting that the inhibitory effect of miR‐485‐5p on lung cancer cell proliferation may be mediated by both senescence and apoptosis. Of note, miR‐485‐5p mimic in HEK293T and HUVEC promoted apoptosis and inhibited proliferation as well (Fig. S8A–D).

### 3.6 *CDK16* KD leads to reduced expression of *MYC* and PD‐L1

**Fig. 6:**
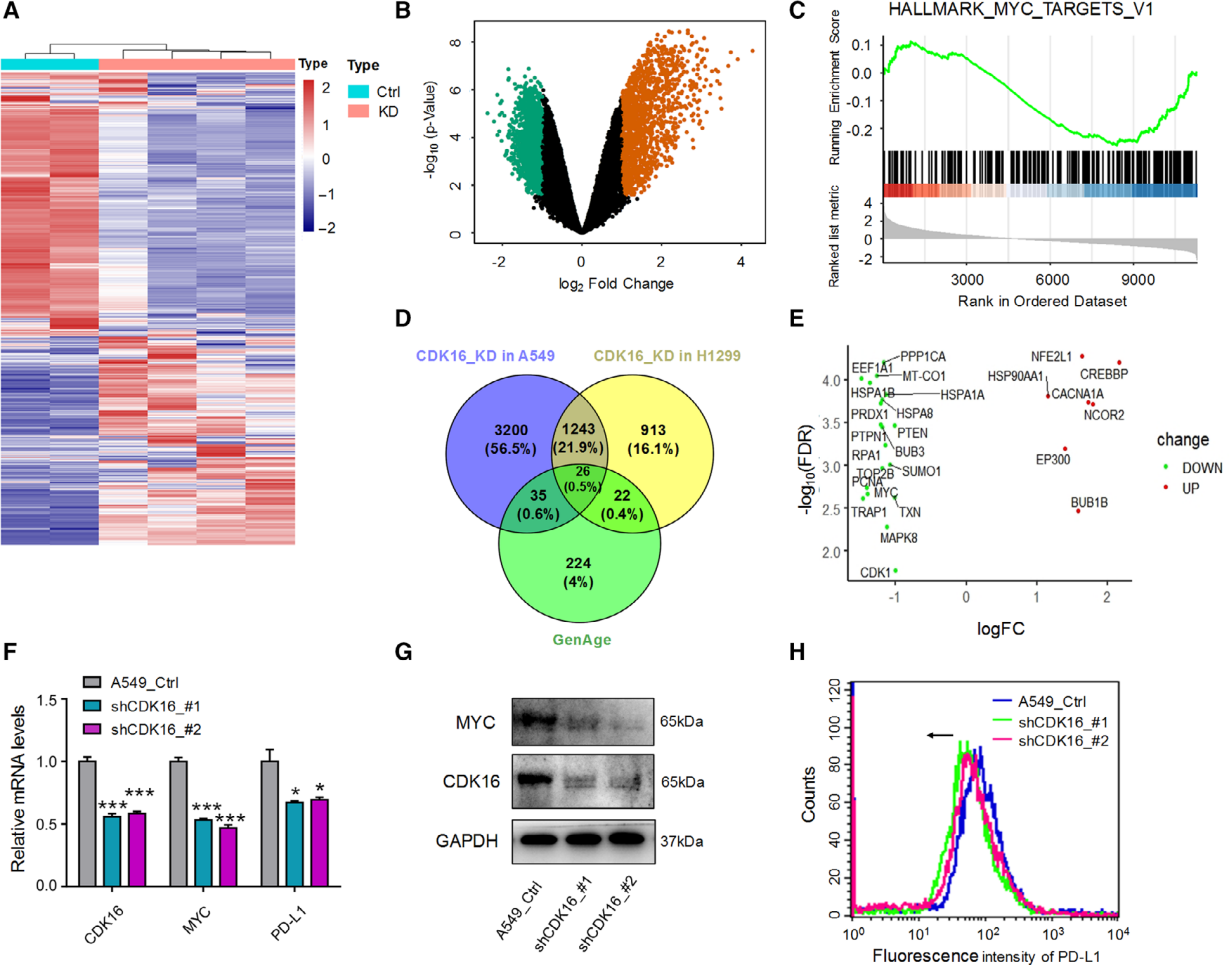
*CDK16* KD leads to reduced *MYC* and membranous PD‐L1 expression in lung cancer cells. (A) Heatmap showing the DEGs between *CDK16*‐KD and control (Ctrl) A549 cells, each sample has two technical replicates. (B) Volcano map showing up‐ and down‐regulated genes in *CDK16*‐KD A549 cells. (C) GSEA using H collection (Hallmark gene sets) to display the enrichment of down‐regulated genes above targeted by MYC. (D) Venn diagram showing the relationship between DEGs in *CDK16‐KD* A549 (or H1299) cells and the human senescence‐associated genes archived in the GenAge database (https://genomics.senescence.info/genes/) [73]. (E) Differential expression analysis of 26 overlapping genes. Up‐regulated (UP) and down‐regulated (DOWN) genes were marked respectively according to their fold change (FC) in A549 cells. (F) qRT‐PCR to detect the reduced expression of *MYC* and *PD‐L1* in *CDK16*‐KD A549 cells. *GAPDH* was used as the internal control. *, *** represent *P* < 0.05 and *P* < 0.001 based on *t‐test* with three independent replicates. Error bars indicated mean ± SEM. (G) Western blot to test the decreased MYC protein expression upon *CDK16*‐KD in A549 cells. GAPDH serves as the internal control. (H) Flow cytometry showing the membranous expression of PD‐L1 on the cell surface of *CDK16*‐KD A549 cells based on three independent replicates. The arrow denotes a decrease in membranous PD‐L1 expression.

We next explored the downstream targets involved in lung cancer cell senescence induced by *CDK16*‐KD. For this purpose, transcriptome‐wide comparison between *CDK16*‐KD and control lung cancer cells (A549 and H1299) was performed using RNA‐seq. Although A549 and H1299 cell lines showed distinct pattern of DEGs caused by *CDK16*‐KD, GSEA showed that downregulated DEGs in these two cell lines shared similar pathway terms, such as apoptosis, p53 pathway, and MYC target genes (Fig. 6A–C; Fig. S9A–C), suggesting that common factors may underlie the *CDK16*‐KD‐induced senescence between A549 and H1299. To examine this, we compared respectively the DEGs in *CDK16‐*KD A549 and H1299 cells with aging‐related genes recorded in the GenAge and CellAge databases in the Human Ageing Genomic Resources [73, 74, 75] and found a total of 26 and 22 known aging‐related genes, respectively (Fig. 6D,E; Figs S9D and S10A). Among them, the proto‐oncogene *MYC* (also known as *c‐MYC*) attracts our attention, since *MYC* activation contributes to the occurrence of diverse cancers, and *MYC* inhibition induces senescence in a variety of cancer cells [50, 76]. Interestingly, *MYC* showed reduced expression in *CDK16*‐KD A549 and H1299 cells as assessed by RNA‐seq (Fig. 6E, Figs S9D and S10B,C) and further validated by qRT‐PCR and Western blot (Fig. 6F,G, Fig. S9E,F). These data suggested that *MYC* may be a possible factor mediating *CDK16*‐KD‐induced senescence. Consistent with the fact that *MYC* serves as a widespread transcription factor that can regulate tumor‐specific gene expression [77], we also found the decreased expression of its target gene *PD‐L1* in both *CDK16*‐KD lung cancer cells (Fig. 6F, Fig. S9E). It has been reported that *MYC* repression downregulates PD‐L1 expression and activates the antitumor immune response [78]. In our study, PD‐L1 protein expression was also detected on the surface of *CDK16*‐KD A549 and H1299 cells, and *CDK16* deficiency caused a decreased PD‐L1 expression on the membrane of A549 and H1299 cells (Fig. 6H; Fig. S9G), suggesting that cellular senescence caused by *CDK16*‐KD might enhance the immune response by inhibiting the MYC/PD‐L1 signaling axis. In summary, *CDK16* KD leads to a reduced expression of *MYC* and PD‐L1, both benefiting antitumor effects.

## 4 Discussion

Understanding CDK regulation has important biomedical implications, a well‐known example of this gene family is that pharmacologic CDK4/6 inhibitors have shown their ability to fight against several solid tumors by inducing cancer cell apoptosis or senescence [10, 11, 12, 26]. The present study revealed that *CDK16* has a new function of inducing senescence in lung cancer cells. *CDK16* is also the only CDK member with APA regulation. APA‐mediated 3′UTR length changes showed opposite trends between cancer (shortening) and senescence (lengthening), accompanying the up‐ and down‐regulation of *CDK16* gene expression, respectively. The less protein production from longer *CDK16* transcript using the distal pA site compared with the shorter one using the proximal pA site can be explained, at least in part, by the specific binding of miR‐485‐5p to the alternative 3′UTR. Moreover, *CDK16* inhibition downregulated *MYC* and PD‐L1 in NSCLC cells, suggesting that the enhanced antitumor immune response may be a downstream effect of inducing cancer cell senescence to exercise its tumor‐suppressive function. The present study demonstrated that APA‐mediated 3′UTR length regulation exists in *CDK16*, a member of the well‐known CDK family, and plays a role in lung cancer cell senescence.

Although senescent cells do not proliferate, they remain metabolically active and can produce secreted proteins with tumor‐suppressing or tumor‐promoting activities [79]. Of note, inducing cancer cell to senescence is becoming a tumor‐suppressing strategy in addition to apoptosis [80]. For example, the cancer preventing ability of p53, a protein capable of initiating apoptosis or senescence, predominantly depends on senescence induction [81], implying that triggering cancer cell to senescence plays important roles in tumor suppression. *CDK16* has been reported to play an oncogenic role in NSCLC by inhibiting apoptosis in a p27‐dependent manner [16]. It also inhibits the production of reactive oxygen species (ROS) and DNA damage response in lung cancer by phosphorylating p53 [17]. Recently, CDK16/Cyclin Y complex has been found necessary for MAPK‐dependent autophagy activation [82, 83, 84]. Although the above evidence builds the link between CDK16 and apoptosis, whether *CDK16* plays a role in senescence remains elusive. The present study demonstrated for the first time that *CDK16* downregulation can induce senescence in both NSCLC and normal cells, suggesting its regulatory role in both physiological and pathological situations.

Alternative polyadenylation and miRNA‐mediated gene silencing can coordinate to participate in post‐transcriptional regulation of gene expression [85]. For example, progressive 3′UTR lengthening caused by APA during embryonic development can significantly enhance the effects of miRNA targeting, since miRNA target sites located in alternative 3′UTRs are more suitable for miRNA binding than those in constructive 3′UTRs [86]. In contrast, the widespread shortening of 3′UTR in cancer cells protects most proto‐oncogenes from miRNA‐mediated inhibition, thereby contributing to the oncogene activity maintenance [35]. Our previous study found that more genes preferred the longer 3′UTR than the shorter one in replicative cellular senescence, and some RBP (TRA2B) bound to the alternative 3′UTR of the target gene (*RRAS2*) to regulate its gene expression and ultimately leading to senescence‐associated phenotypes [33]. However, whether APA‐mediated 3′UTR length changes can combine with miRNA recognition to regulate cancer cell senescence is unclear. The present study discovered the first example of miR‐485‐5p binding to the alternative *CDK16* 3′UTR and in turn reducing the protein production of corresponding isoform. *CDK16*‐KD and miR‐485‐5p overexpression can both lead to senescence‐associated phenotypes in cancer cells. Noteworthy, miR‐485‐5p has been considered to be a tumor‐suppressive miRNA in multiple cancer types, and its decreased levels have been found in many cancer tissues and cancer cell lines [87]. In addition, miR‐485‐5p can inhibit the growth and invasion of NSCLC, and its low expression is significantly associated with poor prognosis [88], suggesting that miR‐485‐5p can be used as a target for cancer therapy in NSCLC. Since one miRNA can target multiple genes, and one gene can be regulated by different miRNAs, it is necessary to figure out the complete miRNA–target interaction network for exploring the potential new therapeutic targets. The present study found that miR‐485‐5p can target a new gene *CDK16*, whose reduced expression promoted senescence‐associated phenotypes. Taken together, a possible deduction can be reached that *CDK16* 3′UTR shortening allows lung cancer cells to escape senescence fate by avoiding miR‐485‐5p targeting on its alternative 3′UTR. This novel molecular finding indicates a potential new target for cancer treatment, though deserves further investigation.

The role of cellular senescence in cancer prevention has been widely investigated [23, 89]. The present study found that *CDK16*‐KD can induce cancer cells to senescence. However, the detailed mechanism of how this process exerts its tumor‐suppressive function still remains not fully understood. Considering that one hallmark of cancer cells is the ability to evade immune surveillance [90] and that one contribution of senescent cells is to interact with immune cells to promote the immunoclearance effect of tumor cells [91], it is valuable to explore whether anti‐tumor immune response increases upon *CDK16* KD. Noteworthy, *CDK16*‐KD led to reduced expression of *MYC* and membranous PD‐L1. MYC seems to be one of the most important carcinogenic factor in human tumorigenesis [92], and it can act as a transcription factor to promote a wide range of gene expression and play a key role in a variety of tumor processes, including immune escape, invasion, and proliferation [77, 90, 93, 94]. *MYC* inactivation can also trigger senescence in various cancer types and promote cancer elimination [50, 95, 96]. Consistently, in the present study, *CDK16*‐KD led to decreased *MYC* expression and senescence of cancer cells. In addition, MYC can directly bind to the promoter of *PD‐L1* gene, and MYC inactivation leads to reduced PD‐L1 mRNA and protein abundance in tumor cells, which in turn enhances the cellular anti‐tumor immune response [78]. The treatment with PD‐L1 antibody has achieved great success in preclinical models and clinical NSCLC therapy, with a satisfied safety profile and controllable side effects [97, 98, 99]. This cooperation between MYC inactivation and immune checkpoint blockade can effectively reverse immune escape and treat lung cancer [78, 100, 101]. Therefore, *CDK16*‐KD‐induced downregulation of membranous PD‐L1 may prevent cancer cells from evading immune recognition and thus benefit patient survival. Our findings suggest that understanding the mechanisms of cancer cell senescence and the way by which senescent cancer cells can be recognized by immune system is crucial for prosenescence cancer therapy. Therefore, *CDK16*, whose downregulation results in cancer cell senescence can serve as a potential novel target for cancer treatment.

## 5 Conclusions

In conclusion, the present study found an opposite trend of the expression and APA usage in *CDK16* between lung cancer and senescent cells. The APA‐mediated 3′UTR shortening of *CDK16* in lung cancer cells enable escaping from miR‐485‐5p binding and consequent transcript degradation. This study demonstrated for the first time that both *CDK16* inhibition and miR‐485‐5p overexpression can induce senescence in lung cancer cells, indicating their potential anticancer capacities. Besides, this study also indicated that surveillance and elimination of senescent cancer cells by immune system might be an alternative strategy for effective cancer therapy.

## Conflict of interest

The authors declare no conflict of interest.

## Author contributions

TN, GW, and QJ designed the study, QJ, BX, and LH performed the experiments, TN, GW, ZZ, and QJ analyzed and interpreted the data, TN, GW, and QJ wrote the manuscript. All authors approved the final version of the manuscript.

## Supporting information


**Fig. S1.** APA events of *CDK16* demonstrated by the relative usage of distal pA site.

**Fig. S2.** 3′UTR shortening of *CDK16* illustrated by RNA‐seq track in LUAD and LUSC.

**Fig. S3.** Down‐regulation of *CDK16* induces senescence in HEK293T and HUVEC.

**Fig. S4.** *CDK16* gene structure annotated by UCSC Genome Browser.

**Fig. S5.** The GFP protein expression in four cells transfected with constructs fused with different 3′UTR length.

**Fig. S6**. Determination of the inhibitory element located in the alternative 3′UTR of *CDK16* near the proximal pA site.

**Fig. S7.** miR‐485‐5p targeting *CDK16* to regulate its gene expression.

**Fig. S8.** miR‐485‐5p induces apoptosis in HEK293T and HUVEC.

**Fig. S9.** *CDK16* knockdown leaded to reduced MYC and membranous PD‐L1 expression in H1299 cells.

**Fig. S10.** The DEGs in *CDK16*‐KD cells overlap with aging‐related genes in CellAge.

**Table S1.** Sequences of primers used in the present study.

## Data Availability

The data that support the findings of this study are available in reference number [61, 62] of this article.
