# Supplementary figures and images for: Lung cancer cells expressing a shortened CDK16 3′UTR escape senescence through impaired miR‐485‐5p targeting
Source: Mol Oncol. 2021 Nov 9;16(6):1347–64. doi: 10.1002/1878-0261.13125 (PMC8936527; doi:10.1002/1878-0261.13125)

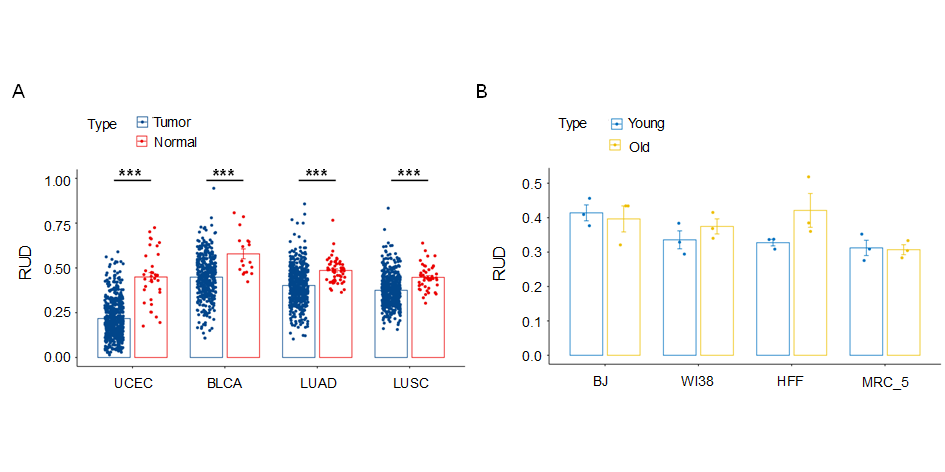

Supplement: Supplementary file 1 — Fig. S1. APA events of CDK16 demonstrated by the relative usage of distal pA site. [file MOL2-16-1347-s012.tif]

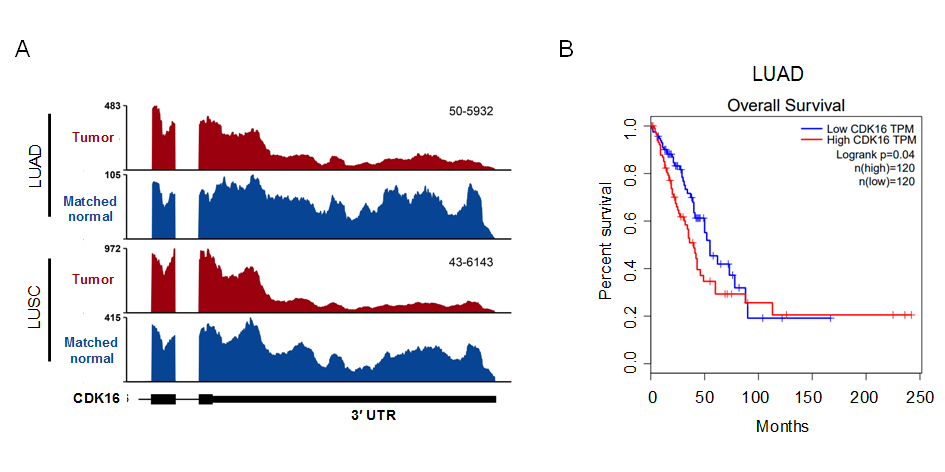

Supplement: Supplementary file 2 — Fig. S2. 3′UTR shortening of CDK16 illustrated by RNA‐seq track in LUAD and LUSC. [file MOL2-16-1347-s003.tif]

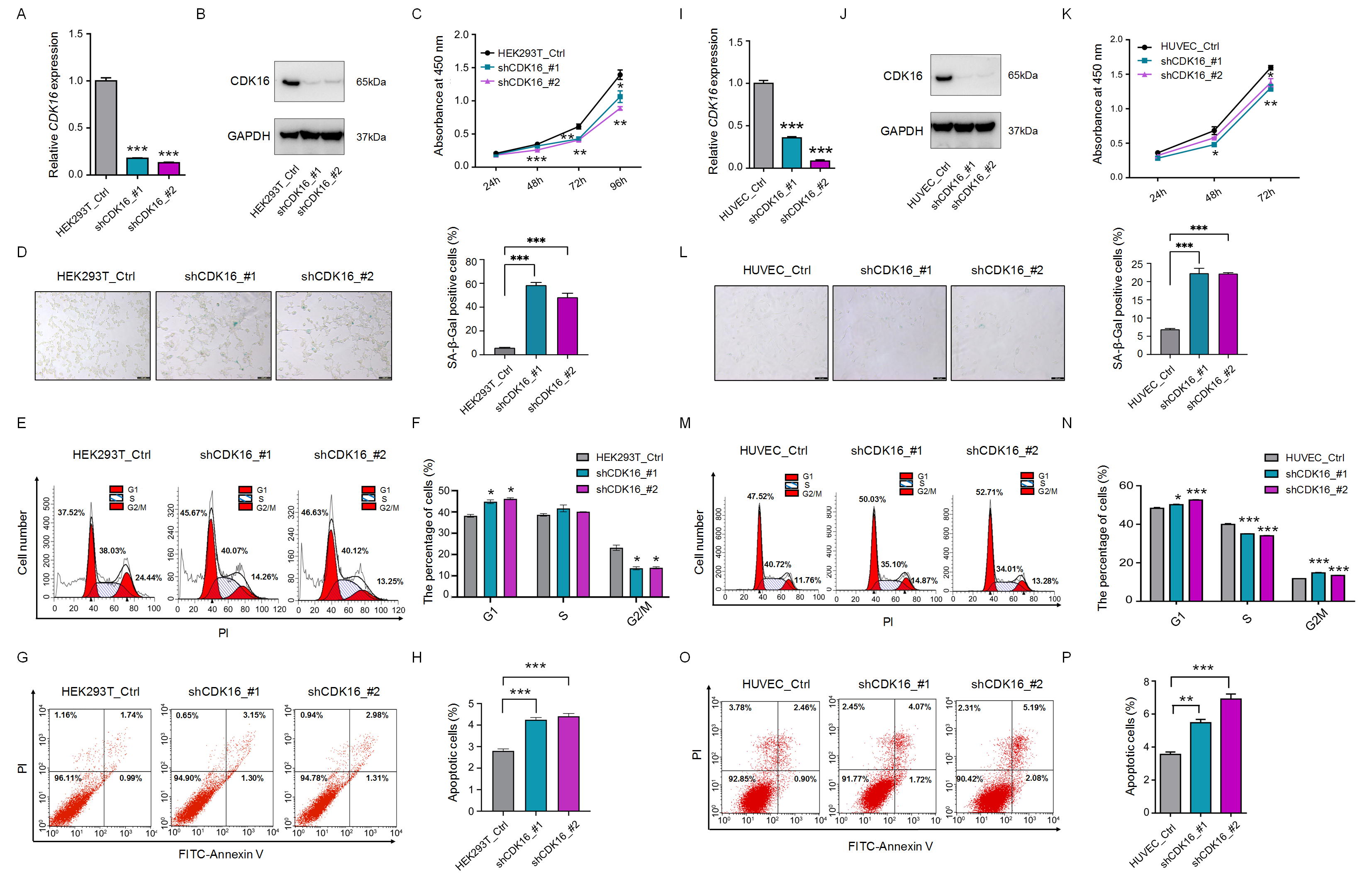

Supplement: Supplementary file 3 — Fig. S3. Down‐regulation of CDK16 induces senescence in HEK293T and HUVEC. [file MOL2-16-1347-s002.tif]

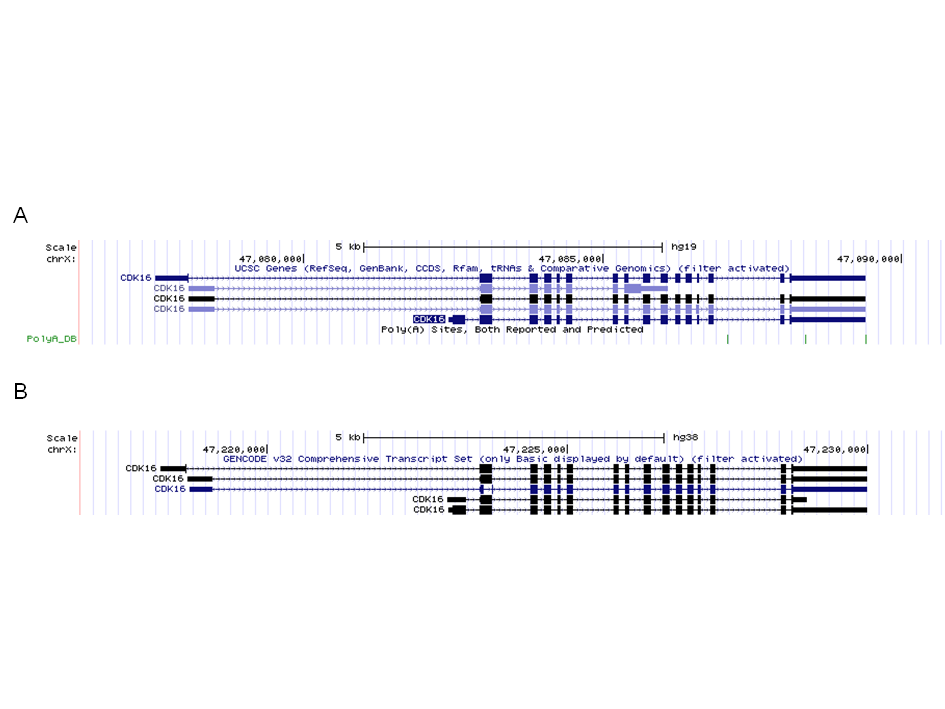

Supplement: Supplementary file 4 — Fig. S4. CDK16 gene structure annotated by UCSC Genome Browser. [file MOL2-16-1347-s004.tif]

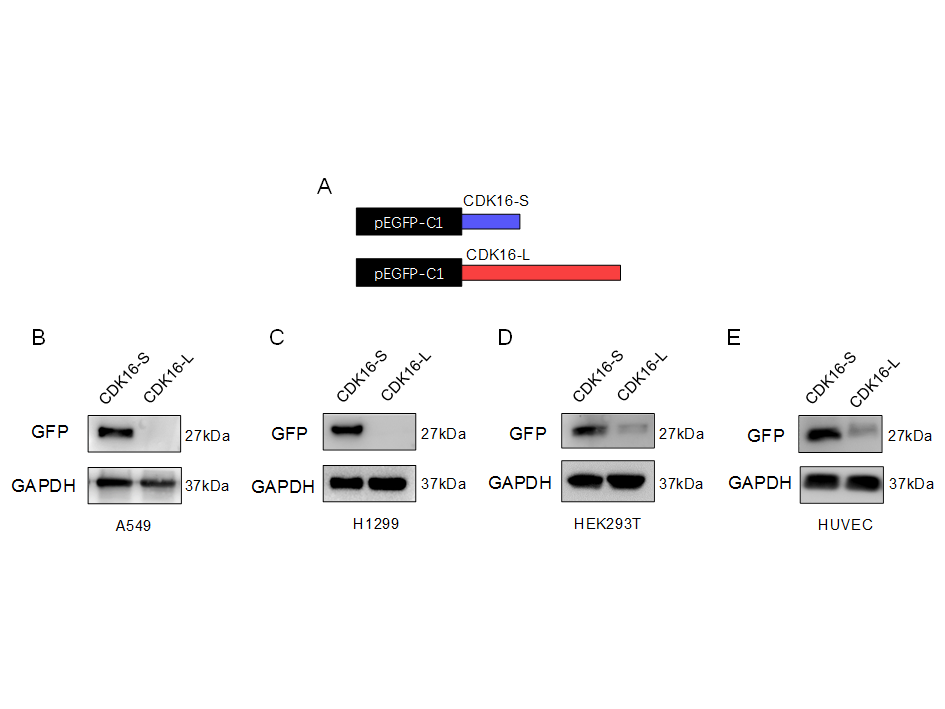

Supplement: Supplementary file 5 — Fig. S5. The GFP protein expression in four cells transfected with constructs fused with different 3′UTR length. [file MOL2-16-1347-s010.tif]

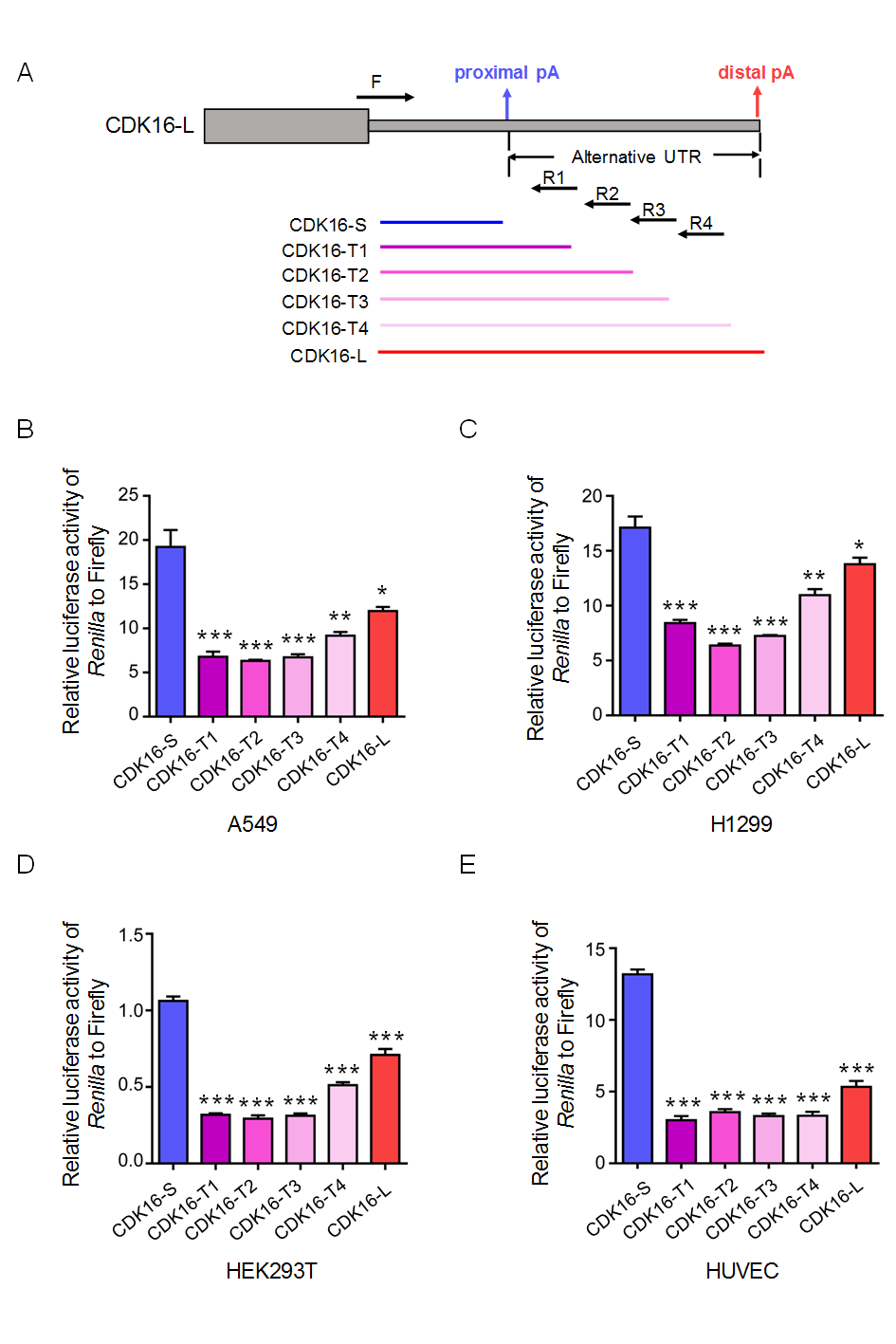

Supplement: Supplementary file 6 — Fig. S6. Determination of the inhibitory element located in the alternative 3′UTR of CDK16 near the proximal pA site. [file MOL2-16-1347-s006.tif]

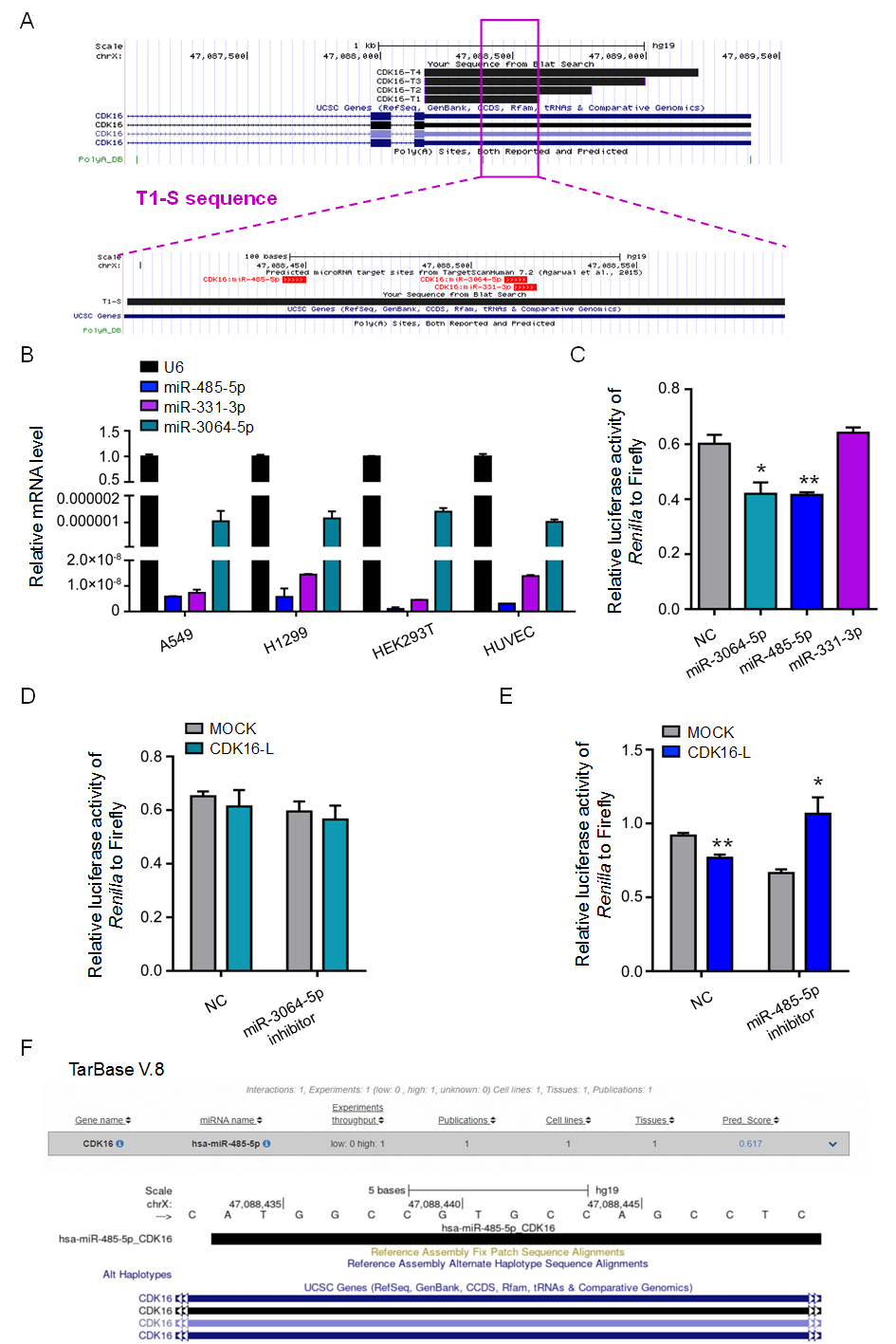

Supplement: Supplementary file 7 — Fig. S7. miR‐485‐5p targeting CDK16 to regulate its gene expression. [file MOL2-16-1347-s007.tif]

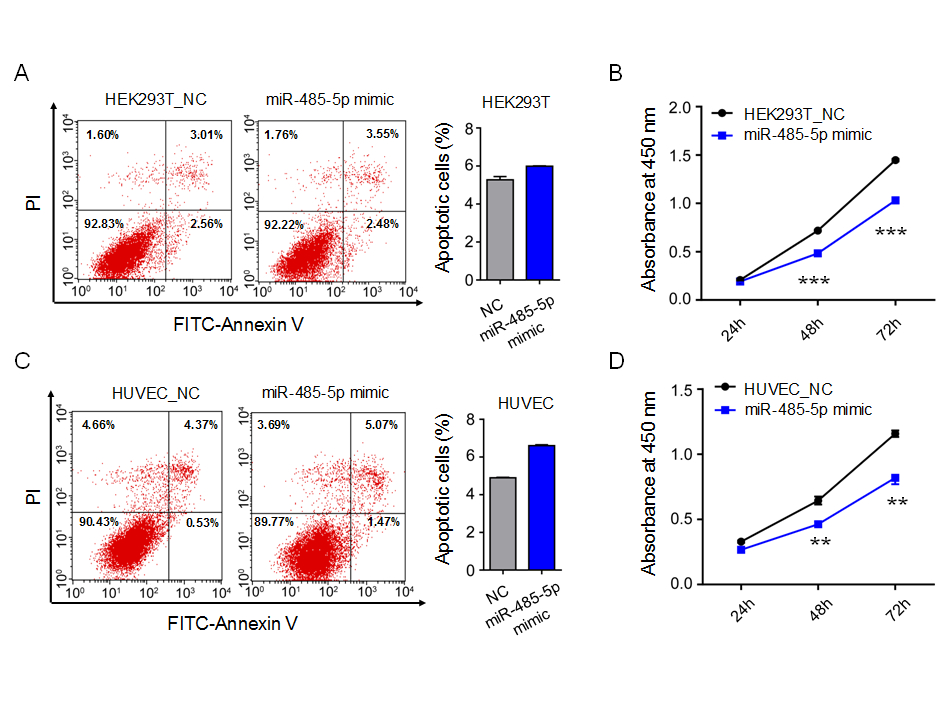

Supplement: Supplementary file 8 — Fig. S8. miR‐485‐5p induces apoptosis in HEK293T and HUVEC. [file MOL2-16-1347-s009.tif]

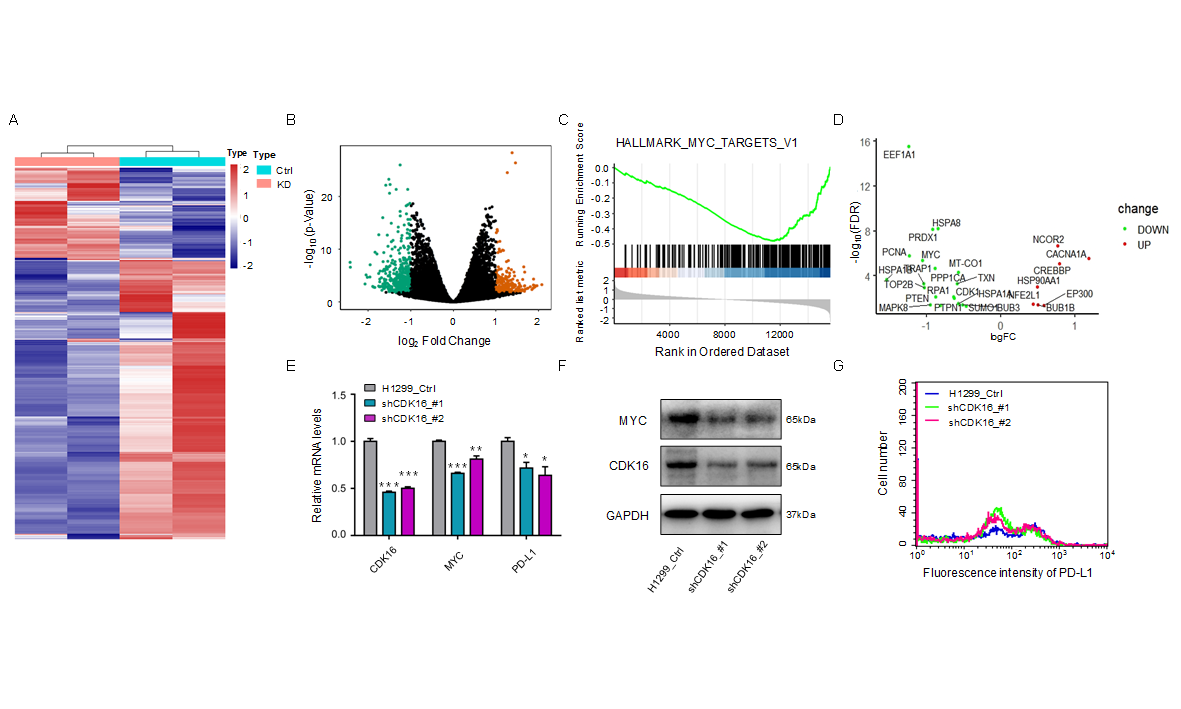

Supplement: Supplementary file 9 — Fig. S9. CDK16 knockdown leaded to reduced MYC and membranous PD‐L1 expression in H1299 cells. [file MOL2-16-1347-s001.tif]

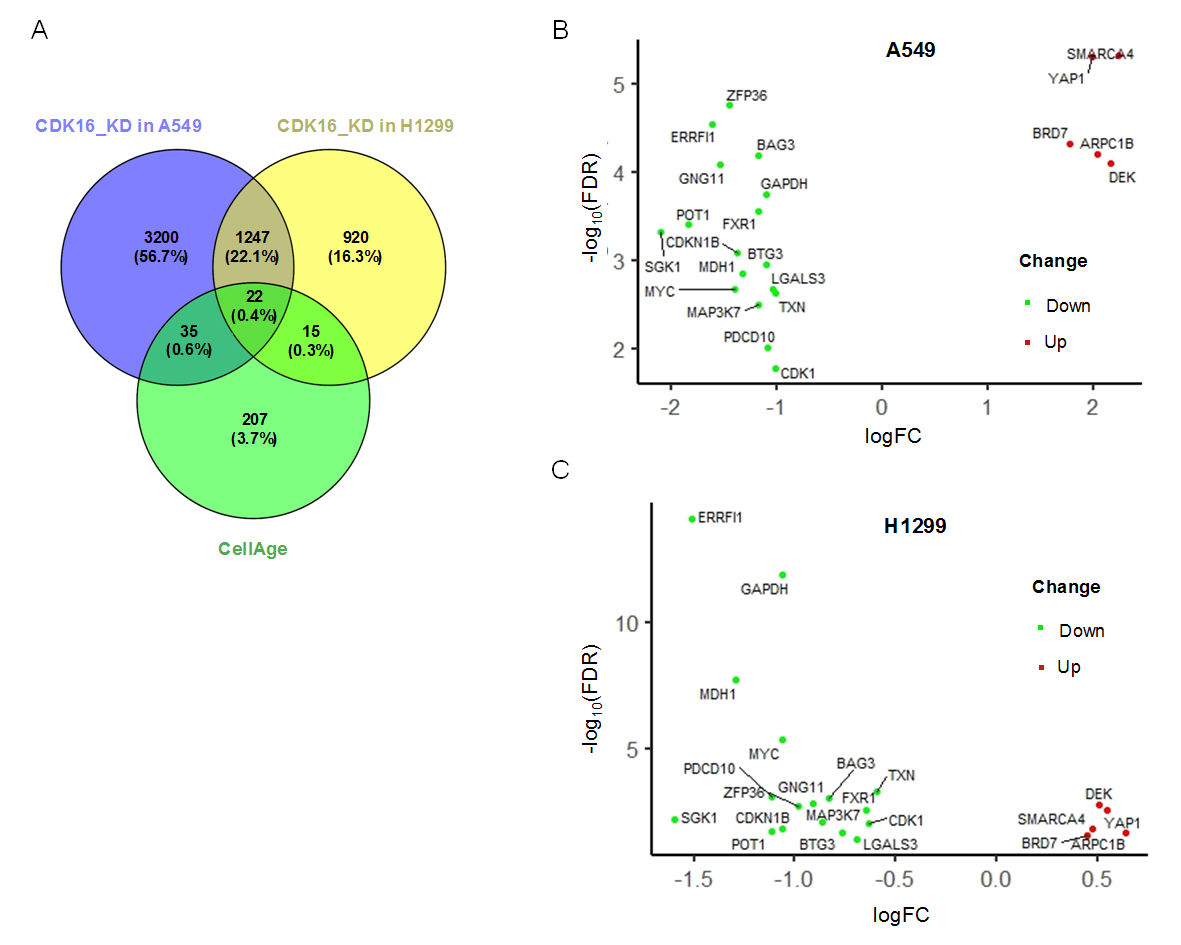

Supplement: Supplementary file 10 — Fig. S10. The DEGs in CDK16‐KD cells overlap with aging‐related genes in CellAge. [file MOL2-16-1347-s005.tif]
