# Supplementary material for: Lung cancer cells expressing a shortened CDK16 3′UTR escape senescence through impaired miR‐485‐5p targeting
Source: Mol Oncol. 2021 Nov 9;16(6):1347–64. doi: 10.1002/1878-0261.13125 (PMC8936527; doi:10.1002/1878-0261.13125)
Supplement: Supplementary file 11 — Table S1. Sequences of primers used in the present study. [file MOL2-16-1347-s008.docx]

**Supplementary Table S1. Sequences of primers used in the present study.**

| primer | Sequence (5′-3′) |
| --- | --- |
| Hq_GAPDH_F | TGGAAGGACTCATGACCACA |
| Hq_GAPDH_R | TTCAGCTCAGGGATGACCTT |
| Hq_CDK16_F | CACCAGAGATTGTGCACGAG |
| Hq_CDK16_R | TTGAGGGTCAGCTTCTCCAG |
| Hq_CDK16-S_F | CTGGAGGGATGCCACACC |
| Hq_CDK16-S_R | CAATGGGTGGGTTGACAGG |
| Hq_CDK16-L_F | AGCCTTTCAGAGACAGGGAC |
| Hq_CDK16-L_R | TGCAGGAGAGAAAGAGTGGG |
| miR-485-5p-RT-primer | GTCGTATCCAGTGCAGGGTCCGAGGTATTCGCACTGGATACGACGAATTC |
| miR-331-3p-RT-primer | GTCGTATCCAGTGCAGGGTCCGAGGTATTCGCACTGGATACGACTTCTAG |
| miR-3064-5p-RT-primer | GTCGTATCCAGTGCAGGGTCCGAGGTATTCGCACTGGATACGACTTGCAC |
| U6-RT-primer | CGCTTCACGAATTTGCGTGTCAT |
| miRNA-qPCR-R | AGTGCAGGGTCCGAGGTATT |
| miR-485-5p-qPCR-F | CGAGAGGCTGGCCGTGAT |
| miR-331-3p-qPCR-F | CGGCCCCTGGGCCTATC |
| miR-3064-5p-qPCR-F | CGCGTCTGGCTGTTGTGGT |
| U6-qPCR-F | GCTTCGGCAGCACATATACTAAAAT |
| U6-qPCR-R | CGCTTCACGAATTTGCGTGTCAT |
