# Supplementary material for: Lung cancer cells expressing a shortened CDK16 3′UTR escape senescence through impaired miR‐485‐5p targeting
Source: Mol Oncol. 2021 Nov 9;16(6):1347–64. doi: 10.1002/1878-0261.13125 (PMC8936527; doi:10.1002/1878-0261.13125)
Supplement: Supplementary file 12 [file MOL2-16-1347-s011.docx]

**Supplementary Figure Legends**

**Supplementary Figure S1.** **APA events of *CDK16* demonstrated by the relative usage of distal pA site.**

(**A**) The preference of proximal pA site in four cancer types (UCEC, BLCA, LUAD, and LUSC). The 3′UTR shortening of *CDK16* in tumors compared to matched normal tissues was illustrated by RUD (Relative Usage of Distal pA site) based on TCGA RNA-seq datasets. Error bars indicated mean ± SEM. (**B**) The RUD value of *CDK16* between young and old cells in four cell types (BJ, WI38, HFF, and MRC_5) based on the public RNA-seq data. Error bars indicated mean ± SEM.

**Supplementary Figure S2. 3'UTR shortening of *CDK16* illustrated by RNA-seq track in LUAD and LUSC.**

(**A**) The preference of proximal polyA site of *CDK16* in LUAD and LUSC tumor samples compared with matched normal samples based on RNA-seq density plot in corresponding TCGA datasets. (**B**) The overall survival curve analysis of LUAD patients with high and low *CDK16* expression based on TCGA RNA-seq data in GEPIA [1].

**Supplementary Figure S3.** **Down-regulation of *CDK16* induces senescence in HEK293T and HUVEC.**

(**A-B**) Validation of *CDK16* knockdown (KD) in HEK293T by qRT-PCR (**A**) and Western blot (**B**). (**C**) Cell proliferation rate evaluated by CCK-8 (Cell Counting Kit-8) assay in *CDK16*-KD HEK293T cells. (**D**) SA-β-Gal (senescence-associated β-galactosidase) staining in *CDK16*-KD HEK293T cells (Left) and the quantitative statistics of positive staining cells (Right). Scale bars = 200 μm. *** represents p < 0.001 based on *t*-test with three independent countings. (**E-F**) Cell cycle analysis in *CDK16*-KD HEK293T cells. Cell cycle arrested at G1 phase in *CDK16*-KD cells was shown with flow cytometry distribution (**E**) and statistical graph of cells at each stage (**F**). (**G-H**) Cell apoptosis assessed by double-staining Annexin V and PI in *CDK16*-KD HEK293T cells. The proportion of cells in each quadrant was obtained by flow cytometry (**G**), and the total Annexin V-positive cells were counted as apoptotic cells (**H**). (**I-J**) Validation of *CDK16* knockdown in HUVEC cells by qRT-PCR (**I**) and Western blot (**J**). (**K**) Cell proliferation rate evaluated by CCK-8 assay in *CDK16*-KD HUVEC cells. (**L**) SA-β-Gal staining in *CDK16*-KD HUVEC. The positive staining cells were considered as senescent cells. Scale bars = 200 μm. *** represents p < 0.001 based on *t*-test with three independent countings. (**M-N**) Cell cycle analysis on *CDK16*-KD HUVEC. Cell cycle arrested at G1 phase in *CDK16*-KD cells was measured by flow cytometry, shown as the distribution (**M**) and statistical graph (**N**) of cells of each cell phase. (**O-P**) Apoptosis analysis by double-staining Annexin V and PI in *CDK16*-KD HUVEC cells. The proportion of cells in each quadrant was determined by flow cytometry (**O**), and the total Annexin V-positive cells were counted as apoptotic cells (**P**). For each panel, error bars indicated mean ± SEM. *, ** and *** stand for p < 0.05, p < 0.01 and p < 0.001, respectively, based on *t*-test with three biological or independent replicates.

**Supplementary Figure S4. *CDK16* gene structure annotated by UCSC Genome Browser.**

(**A**) RefSeq gene annotation of *CDK16* in the version of human genome 19 (hg19). Poly(A) sites predicted by PolyA_DB [2] were shown in the UCSC Genome Browser. (**B**) RefSeq gene annotation of *CDK16* in the version of human genome 38 (hg38).

**Supplementary Figure S5.** **The GFP protein expression in four cells transfected with constructs fused with different** **3′UTR length.**

(**A**) Schematic diagram of plasmid construction. Short and long 3′UTR sequence of *CDK16* was cloned into the 3′ end of pEGFP-C1 vector, respectively.

(**B-E**) Western blot to detect the fused GFP protein expression in A549 (**B**), H1299 (**C**), HEK293T (**D**), and HUVEC cells (**E**). Significantly decreased GFP expression was observed in four cells transfected with pEGFP-C1 plasmid fused with long 3′UTR sequence (CDK16-L) compared with the construct fused with short one (CDK16-S). GAPDH was used as the internal control in each cell type.

**Supplementary Figure S6. Determination of the inhibitory element located in the alternative 3′UTR of *CDK16* near the proximal pA site.**

(**A**) Schematic map illustrating the primer design for constructing four plasmids containing successively truncated CDK16-L. The proximal (Blue) and distal (Red) pA sites were marked with vertical arrows at the corresponding positions in the 3′UTR. PCR was performed with the forward primer (F) and corresponding reverse primers (R1, R2, R3, R4) to obtain the truncated 3′UTR of different length. (**B**-**E**) Dual-luciferase reporter assay to test the influence of 3′UTR length (CDK16-S, CDK16-T1, CDK16-T2, CDK16-T3, CDK16-T4 and CDK16-L) on the luciferase activity in A549 (**B**), H1299 (**C**), HEK293T (**D**), and HUVEC cells (**E**). Relative luminescence of *Renilla* luciferase was normalized using the reference Firefly luciferase activity. *, **, and *** represents p < 0.05. p < 0.01, and p < 0.001 respectively based on *t*-test with four biological replicates. Error bars indicated mean ± SEM.

**Supplementary Figure S7. miR-485-5p targeting *CDK16* to regulate its gene expression.**

(**A**) Prediction of possible regulatory miRNAs in the 3′UTR sequence between CDK16-T1 and CDK16-S (T1-S) by TargetScan. Four 3′UTR fragments of different length (CDK16-T1, CDK16-T2, CDK16-T3, CDK16-T4) were cloned into the psiCHECK2 vector, followed by Sanger sequencing and mapping to the human genome (hg19), as illustrated by UCSC Genome Browser (Up). miRNAs binding sites in T1-S sequence was predicted by TargetScan (Down). (**B**) RT-qPCR to detect the endogenous expression of three miRNAs in four cell types (A549, H1299, HEK293T, and HUVEC), *U6* was used as the internal control. Error bars indicated mean ± SEM. (**C**) Dual-luciferase reporter assay to test the effect of miRNA mimics on the luciferase activity of CDK16-L. CDK16-L was co-transfected with miRNA mimics (that of miR-3064-5p, miR-485-5p or miR-331-3p) or negative control (NC) into HEK293T cells. Error bars indicated mean ± SEM. * and ** represent p < 0.05 and p < 0.01 respectively based on *t*-test with four biological replicates. (**D-E**) Dual-luciferase reporter assay to test the effect of miRNA inhibitors on the luciferase activity of CDK16-L. CDK16-L or empty vector (MOCK) was co-transfected with negative control (NC) or miRNA inhibitors (that for miR-3064-5p (**D**) or miR-485-5p (**E**), respectively into HEK293T cells. Error bars indicated mean ± SEM. * and ** represent p < 0.05 and p < 0.01, respectively, based on *t*-test with four biological replicates. (**F**) *CDK16* was predicted as miR-485-5p target by TarBase v8 in DIANA Tools [3]. The graphic of miR-485-5p binding to *CDK16* was visually displayed on the UCSC Genome Browser.

**Supplementary Figure S8. miR-485-5p induces apoptosis in HEK293T and HUVEC.**

(**A**) Apoptosis analysis using double-staining Annexin V and PI in HEK293T cells transfected with miR-485-5p mimic or negative control (NC) and detected by flow cytometer. Total Annexin V-positive cells were considered as apoptotic cells (Right panel). (**B**) Cell proliferation rate evaluated by CCK-8 assay in miR-485-5p-OE HEK293T cells. (**C-D**) Apoptosis detection (**C**), and CCK-8 assay (**D**) performed in HUVEC cells transfected with NC and miR-485-5p mimic. For each panel, error bars indicated mean ± SEM. *, **, and *** represent p <0.05, p < 0.01 and p < 0.001, respectively based on *t-test* with three biological replicates.

**Supplementary Figure S9. *CDK16* knockdown leaded to reduced *MYC* and membranous PD-L1 expression in H1299 cells.**

(**A**) Heatmap showing the expression of differentially expressed genes (DEGs) in control (Ctrl) and *CDK16-*KD (KD) H1299 cells, as shown by the Z-score-transformed FPKM (Fragments Per Kilobase per Million), each sample has two technical replicates. (**B**) Volcano map of up- and down-regulated genes in *CDK16-*KD H1299 cells. (**C**) Gene Set Enrichment Analysis (GSEA) using H collection displaying the enrichment of down-regulated genes targeted by MYC in *CDK16*-KD H1299 cells. (**D**) Differential expression analysis of 26 overlapping genes. Up-regulated (UP) and down-regulated (DOWN) genes were marked respectively according to their fold change (FC) in H1299 cells. (**E**) qRT-PCR to detect the reduced expression of *MYC* and *PD-L1* in *CDK16*-KD H1299 cells. *GAPDH* was used as the internal control. *, **, and *** represent p < 0.05, p < 0.01 and p < 0.001 based on *t-test* with three independent replicates. Error bars indicated mean ± SEM. (**F**) Western blot to validate the decreased MYC protein expression. GAPDH serves as the internal control. (**G**) Flow cytometry analysis showing the membranous expression of PD-L1 on the cell surface of *CDK16*-KD H1299 cells.

**Supplementary Figure S10. The DEGs in *CDK16*-KD cells overlap with aging-related genes in CellAge.**

(**A**) Venn diagram showing the relationship between DEGs (Differentially Expressed Genes) in *CDK16*-KD cells (based on A549 and H1299) and the human senescence-associated genes archived in the GellAge database (https://genomics.senescence.info/cells/)[4]. (**B**-**C**) Differential expression analysis of the 22 overlapping genes. Up-regulated (UP) and down-regulated (DOWN) genes were marked respectively according to their fold change (FC) in *CDK16*-KD A549 (**B**) and *CDK16*-KD H1299 cells (**C**).

**References**

1. Tang, Z., Li, C., Kang, B., Gao, G., Li, C. & Zhang, Z. (2017) GEPIA: a web server for cancer and normal gene expression profiling and interactive analyses, *Nucleic Acids Res.* 45, W98-W102.

2. Zhang, H., Hu, J., Recce, M. & Tian, B. (2005) PolyA_DB: a database for mammalian mRNA polyadenylation, *Nucleic Acids Res.* 33, D116-20.

3. Karagkouni, D., Paraskevopoulou, M. D., Chatzopoulos, S., Vlachos, I. S., Tastsoglou, S., Kanellos, I., Papadimitriou, D., Kavakiotis, I., Maniou, S., Skoufos, G., Vergoulis, T., Dalamagas, T. & Hatzigeorgiou, A. G. (2018) DIANA-TarBase v8: a decade-long collection of experimentally supported miRNA-gene interactions, *Nucleic acids research.* 46, D239-D245.

4. Avelar, R. A., Ortega, J. G., Tacutu, R., Tyler, E. J., Bennett, D., Binetti, P., Budovsky, A., Chatsirisupachai, K., Johnson, E., Murray, A., Shields, S., Tejada-Martinez, D., Thornton, D., Fraifeld, V. E., Bishop, C. L. & de Magalhaes, J. P. (2020) A multidimensional systems biology analysis of cellular senescence in aging and disease, *Genome Biol.* 21, 91.
